# LIM homeodomain transcription factor Isl1 directs normal pyloric development by targeting Gata3

**DOI:** 10.1186/1741-7007-12-25

**Published:** 2014-03-27

**Authors:** Yushan Li, Jirong Pan, Chao Wei, Juan Chen, Ying Liu, Jiali Liu, Xiaoxin Zhang, Sylvia M Evans, Yan Cui, Sheng Cui

**Affiliations:** 1 State Key Laboratory of Agrobiotechnology, College of Biological Sciences, China Agricultural University, Beijing, People’s Republic of China; 2 Skaggs School of Pharmacy, University of California, San Diego, 9500 Gilman DriveLa Jolla, CA 92093, USA; 3 The 306th Hospital of People’s Liberation Army, Beijing, People’s Republic of China

**Keywords:** α-smooth muscle actin, Gata3, Isl1, Pylorus

## Abstract

**Background:**

Abnormalities in pyloric development or in contractile function of the pylorus cause reflux of duodenal contents into the stomach and increase the risk of gastric metaplasia and cancer. Abnormalities of the pyloric region are also linked to congenital defects such as the relatively common neonatal hypertrophic pyloric stenosis, and primary duodenogastric reflux. Therefore, understanding pyloric development is of great clinical relevance. Here, we investigated the role of the LIM homeodomain transcription factor Isl1 in pyloric development.

**Results:**

Examination of Isl1 expression in developing mouse stomach by immunohistochemistry, whole mount *in situ* hybridization and real-time quantitative PCR demonstrated that Isl1 is highly expressed in developing mouse stomach, principally in the smooth muscle layer of the pylorus. Isl1 expression was also examined by immunofluorescence in human hypertrophic pyloric stenosis where the majority of smooth muscle cells were found to express Isl1. *Isl1* function in embryonic stomach development was investigated utilizing a tamoxifen-inducible *Isl1* knockout mouse model. *Isl1* deficiency led to nearly complete absence of the pyloric outer longitudinal muscle layer at embryonic day 18.5, which is consistent with *Gata3* null mouse phenotype. Chromatin immunoprecipitation, luciferase assays, and electrophoretic mobility shift assays revealed that Isl1 ensures normal pyloric development by directly targeting *Gata3*.

**Conclusions:**

This study demonstrates that the Isl1-*Gata3* transcription regulatory axis is essential for normal pyloric development. These findings are highly clinically relevant and may help to better understand pathways leading to pyloric disease.

## Background

The vertebrate gut is a remarkable structure that ingests and digests food, absorbs nutrients, and removes waste products. The gut originates from a simple tubular structure composed of three germ layers including an underlying endoderm, a surrounding splanchnic mesoderm, and an ectoderm [1-3]. In mouse embryos, the gut becomes patterned along the anterior-posterior, dorsal-ventral, left-right, and radial axes. The gut tube consists of the foregut, midgut, and hindgut along its anterior-posterior axis [4,5]. As development progresses, the foregut gives rise to the esophagus, stomach, liver, lungs, and pancreas. The midgut forms the small intestine and the hindgut develops into the large intestine [1,5-8].

The stomach is derived from the posterior foregut. The stomach morphologically differentiates from the foregut tube around embryonic day 9.5 (E9.5) and the expansion of the pre-gastric mesenchyme allows the domain of the stomach to be visible beginning at E10.5 [9]. Mesenchymal cells of stomach differentiate into four distinct concentric layers, including lamina propria, muscularis mucosae, and circular and longitudinal smooth muscle at different stages of embryonic development [10]. By E11.5, the stomach is distinctly enlarged. The stomach smooth muscle differentiates at E13, with a distinct layer of α-smooth muscle actin (α-SMA)-positive cells appearing and a circular muscle layer forming throughout the stomach [11]. The smooth muscle layer thickens in the constricted prospective pyloric sphincter region at about E14.5 [2,9]. At E18.5, the pyloric sphincter begins to function in preventing the reflux of duodenal contents into the stomach [9].

The posterior or pylorus portion of the stomach is the anatomical junction between the stomach and the duodenum. At the terminus of the pylorus, the distinct valvular flaps of the pyloric sphincter can be seen [2]. Under normal physiological conditions, the stomach depends on its peristaltic contraction to grind and thrust the partially digested food, and the pylorus relies on its thickened pyloric sphincter to control the flow of food into the small intestine. Abnormalities in pyloric development or in the contractile function of the pylorus cause reflux of duodenal contents into the stomach and increase the risk of gastric metaplasia and cancer [12,13]. Abnormalities of the pylorus are related to congenital defects [14-16]. Therefore, much attention has been given to the regulating elements and pathways of stomach development, especially pylorus and pyloric sphincter development.

Previous data in chick suggested that bone morphogenetic protein (BMP) signaling regulates mesenchymal expression of *Nkx2.5* and *Sox9*, which affects the character of the pyloric epithelium but has no effect on pyloric smooth muscle [5,17], suggesting that mesenchymal signaling by unknown factors affects the pyloric epithelial phenotype. In the mouse, molecular mechanisms of pyloric formation are little understood, with relatively few of the factors required for normal pyloric development having been identified. Those that have been include *Sox9*[17], *Six2*[9], *Bapx1*[18], *Nkx2.5*[3,17], *Gremlin*[9], and *Gata3*[19,20]. Ablation of the homeodomain transcription factor, *Six2*, expressed in posterior stomach, disrupts thickening of the pyloric smooth muscle layer and attenuates constriction of the pylorus sphincter. In addition, loss of *Six2* eliminates *Sox9* expression, and reduces *Nkx2.5* and *Gremlin* expression in the pylorus, although this expression later recovers [9], suggesting that *Six2*, *Sox9*, *Nkx2.5*, and *Gremlin* are required for pyloric development. In addition, *Nkx2.5*, *Sox9*, and *Gata3* are co-expressed in the dorsal pyloric outer longitudinal muscle (OLM) layer that matures between E14.5 and E16.5. Following deletion of *Nkx2.5* or *Gata3*, the dorsal pyloric OLM is almost absent and constriction of the pylorus sphincter is attenuated [20].

The LIM homeodomain (LIM-HD) transcription factor Isl1 was originally found to function as an insulin gene enhancer binding protein [21]. Isl1 is comprised of two tandem LIM domains and a homeodomain. The homeodomain, with its helix-turn-helix structure, binds to regulatory DNA sequences of target genes, while the LIM domains are mainly involved in protein-protein interactions that regulate the activity of the LIM-HD [22]. Isl1 plays critical roles in cell determination, proliferation, and differentiation in the nervous system [23,24], heart [25], and pituitary gland [26]. In addition, Isl1 expression has been detected in gastric mesenchyme [27,28] and gastrointestinal epithelium in both embryonic and adult mice [29]. However, the role of Isl1 in stomach development has yet to be explored. In the present study, we examined Isl1 expression in the stomach. Isl1 was highly expressed in the posterior stomach in early stages of development and was mainly restricted to the smooth muscle cells of the pylorus. To examine *Isl1* function in stomach development, we utilized a tamoxifen-inducible knockout mouse model. An inducible model was needed because *Isl1-/-* mutants die at approximately E10.5 owing to defects in heart formation. Our results show that Isl1 is vital for formation of the pyloric OLM layer during stomach organogenesis.

## Results

### Isl1 is expressed in embryonic mouse stomach

**Figure 1:**
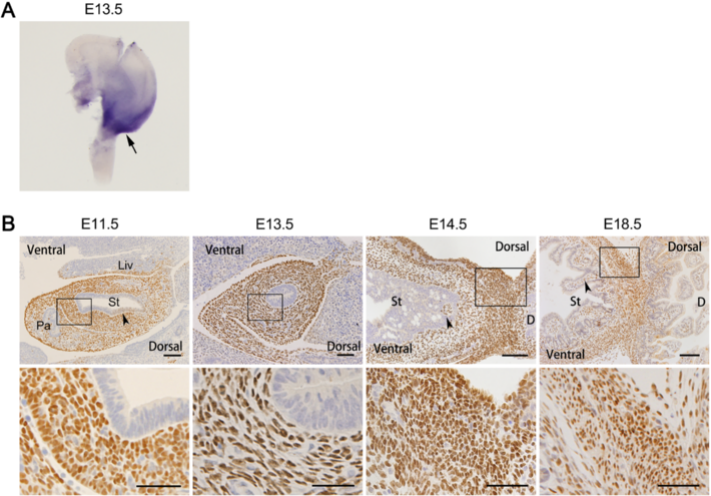
***Isl1 *is expressed in developing mouse stomach. (A)** Embryonic stomach WISH analysis demonstrated that *Isl1* expression was limited to the pylorus at E13.5 (arrow). **(B)** Immunohistochemical staining for Isl1 in stomach. Sections from embryos were arranged in rostral to caudal sequence at E11.5 and E13.5, and Isl1 expression was mainly localized to the mesenchyme of the posterior stomach. At E14.5 and E18.5, Isl1 was highly expressed in smooth muscle cells of the pylorus, although there were some Isl1-positive cells in the lamina propria (arrowheads). Enlarged images in boxed regions are shown below original images. D, duodenum; Liv, liver; Pa, pancreas; St, stomach. Scale bars of original images: 100 μm; scale bars of enlarged images: 50 μm.

We examined *Isl1* mRNA levels in embryonic mouse stomach by real-time quantitative PCR (RT-qPCR) and whole mount *in situ* hybridization (WISH). *Isl1* mRNA was initially detected at E11.5 by RT-qPCR. *Isl1* reached the highest level at E13.5, followed by a sharp decline at E14.5, and had no significant changes into adulthood (Additional file 1: Figure S1a). This result was similar to a previous report [29]. The localization of *Isl1* mRNA expression was investigated using WISH. We performed *Isl1* WISH in embryonic stomach at E11.5, E13.5, and E14.5. At E11.5, *Isl1* was localized to the posterior half of the stomach (Additional file 1: Figure S1b). However, the *Isl1* WISH signal was much stronger and condensed in the pylorus by E13.5 (Figure 1A). As stomach development progressed, the pylorus continued to express *Isl1* and expression of *Isl1* extended to the prospective pyloric sphincter at E14.5 (Additional file 1: Figure S1b). However, the *Isl1* WISH signal weakened considerably from E13.5 to E14.5. These *Isl1* WISH results concurred with *Isl1* RT-qPCR results.

We then assessed Isl1 protein expression by immunohistochemistry. Results demonstrated that Isl1 was mainly localized to the posterior stomach mesenchyme from E11.5 to E13.5, then Isl1 was expressed in smooth muscle cells of the pylorus (Figure 1B and Additional file 1: Figure S1c), and was also detectable in the lamina propria (Figure 1B, arrowheads). In adult mouse stomach, only a few Isl1-positive cells were observed in the smooth muscle layer (Additional file 1: Figure S1c).

### Isl1-positive cells are co-expressed with α-smooth muscle actin in embryonic mouse stomach

**Figure 2:**
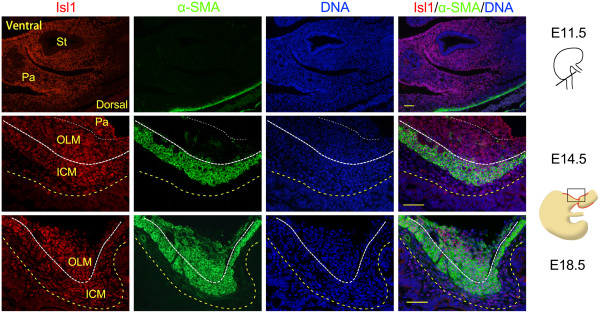
**Double immunostaining for Isl1 and α-smooth muscle actin in mouse smooth muscle cells of the dorsal pylorus.** Isl1 and α-SMA co-expression in smooth muscle cells at E11.5, E14.5, and E18.5. Yellow dotted lines mark the epithelial basement membrane, white thick dotted lines indicate ICM and OLM boundary, and white dotted lines indicate OLM and pancreas boundary. Red staining is Isl1, green staining is α-SMA, and DAPI nuclear counterstaining (DNA) is blue. α-SMA, α-smooth muscle actin; ICM, inner circular muscle; OLM, outer longitudinal muscle; Pa, pancreas; St, stomach. Scale bars: 50 μm.

To see whether Isl1 expression was related to smooth muscle development of the pylorus, we examined the expression of Isl1 and the earliest smooth muscle marker α-SMA using immunofluorescence. Results demonstrated that the proportion of Isl1-positive cells expressing α-SMA gradually increased (Figure 2). At E11.5, no α-SMA-positive cells were detected, although Isl1 was highly expressed in the mesenchyme of the posterior stomach. At E14.5, a subset of Isl1-positive cells, mainly those in the inner circular muscle (ICM) of the pylorus, were α-SMA positive. By E16.5, pyloric OLM has already undergone differentiation [20]. At E18.5, the majority of Isl1-positive cells in the pylorus were α-SMA positive. Isl1 expression persisted in mature pyloric ICM and OLM, and lamina propria cells also expressed Isl1 (Additional file 1: Figure S2). In addition, Isl1 expression was examined in human samples of hypertrophic pyloric stenosis by immunofluorescence, and results demonstrated that Isl1 was also expressed in human smooth muscle cells of the pylorus (Additional file 1: Figure S3). Therefore, these results suggest that Isl1 may participate in the formation of pyloric sphincter.

### Isl1 expression is effectively ablated in *Isl1*^*MCM/F*^-inducible knockout mice

**Figure 3:**
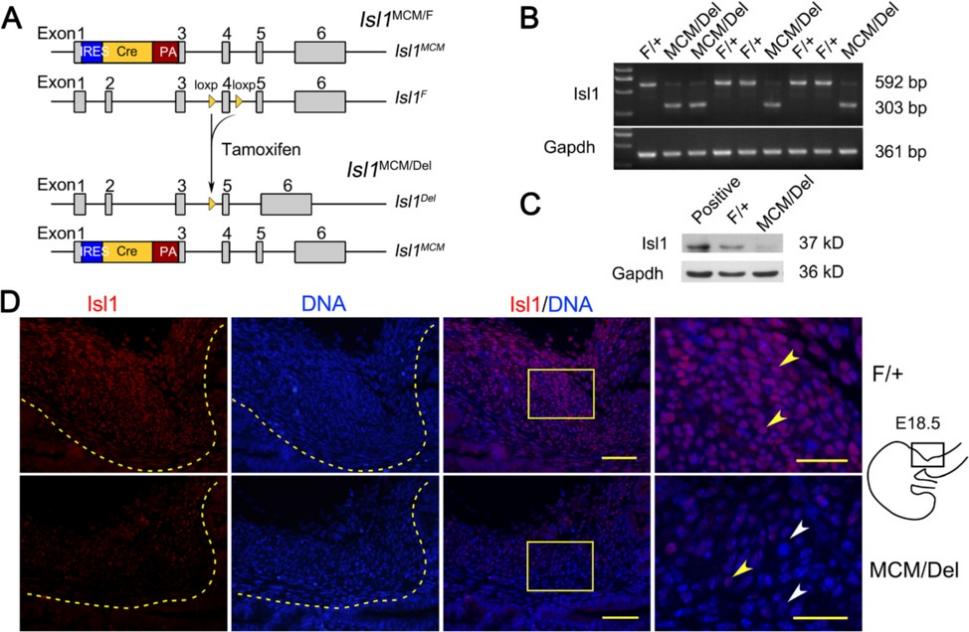
**Efficiency of *Isl1 *ablation in stomachs of *Isl1***^***MCM/Del ***^**mutant mouse stomachs at E18.5. (A)** Tamoxifen-inducible Cre recombinase excised DNA sequences flanked by two *loxP* sites. **(B)***Isl1* RNA levels were ablated in *Isl1*^*MCM/Del*^ mutant stomachs as seen by semi-quantitative PCR. *Isl1*^*F/+m*^ice showed a 592 base pair product whereas *Isl1*^*MCM/Del*^ mice generated a 303 base pair product. **(C)** Isl1 was significantly down-regulated at the protein levels in *Isl1*^*MCM/Del*^ mutant stomachs as shown by western blot. Expression of embryos at E11.5 was used as positive control. **(D)** Isl1 protein expression in *Isl1*^*F/+a*^nd *Isl1*^*MCM/Del*^ embryonic pylorus. Isl1 expression was significantly reduced in *Isl1*^*MCM/Del*^ embryonic stomachs, as seen by immunofluorescence. Images in *Isl1*^*F/+a*^nd *Isl1*^*MCM/Del*^ were processed on the same slide and photographed at the same exposure. Enlarged images of the boxed areas are shown on the right side of the merged pictures. Yellow arrowheads show representative Isl1-positive cells, and white arrowheads show representative Isl1-negative cells. Yellow dotted lines mark the epithelial basement membrane. Scale bars: 50 μm.

To investigate effects of *Isl1* ablation on stomach development, we utilized *Isl1*^*MCM/F*^-inducible Cre (*Isl1*^*MCM/Del*^) mice (Figure 3A) and *Isl1*^*F/+m*^ice were used as controls [30,31]. Embryos were genotyped by PCR at E18.5 (Additional file 1: Figure S4) and intact or mutant *Isl1* mRNA was distinguished by semi-quantitative PCR (Figure 3B).

Western blot analyses showed that Isl1 protein levels in embryonic stomach of *Isl1*^*MCM/Del*^ mice were significantly lower than those in *Isl1*^*F/+m*^ice (Figure 3C). Immunofluorescence results demonstrated significantly reduced Isl1 staining in pylorus of *Isl1*^*MCM/Del*^ mice as compared to controls (Figure 3D). These data demonstrate that *Isl1* expression was effectively down-regulated in *Isl1*^*MCM/Del*^ mutant stomachs.

### Pyloric abnormalities in *Isl1*^*MCM/F*^ mutants

**Figure 4:**
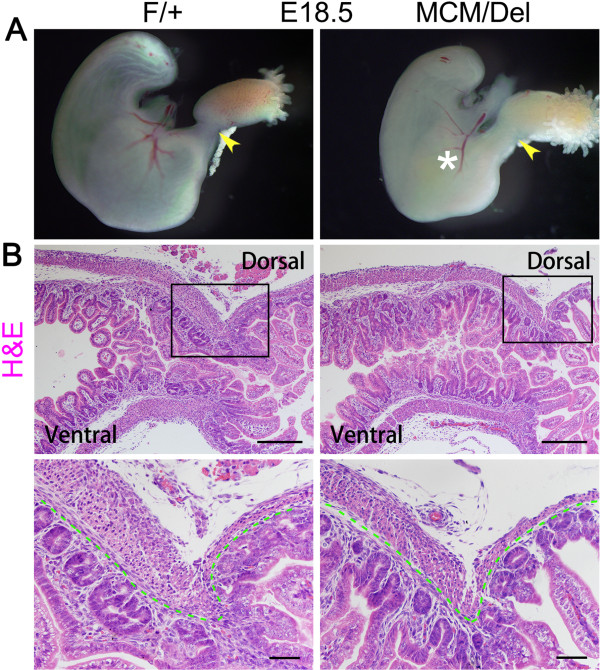
**Morphological and histological changes in developing stomach of *Isl1***^***MCM/Del ***^**mutants. (A)** Gross and microscopic evidence for stomach defects in *Isl1*^*MCM/Del*^ mice. Whole mount views at E18.5 in *Isl1*^*F/+a*^nd *Isl1*^*MCM/Del*^ mouse stomachs. *Isl1*^*MCM/Del*^ mutant stomachs lacked a functional pyloric sphincter (arrowhead), thereby allowing reflux of fluid as observed in mutant embryos. Yellow fluid is denoted by asterisk. **(B)** Hematoxylin and eosin staining of *Isl1*^*F/+a*^nd *Isl1*^*MCM/Del*^ mouse pylorus at E18.5. The dorsal pyloric smooth muscle (black boxed region) was prominent in *Isl1*^*F/+e*^mbryos, but was much thinner in *Isl1*^*MCM/Del*^ embryos. The remainder of the pylorus was histologically normal. Green dotted lines mark the epithelial basement membrane. Enlarged images in boxed regions are shown below original photos. Scale bars of original photos: 200 μm; scale bars of enlarged images: 50 μm. H&E, hematoxylin and eosin.

**Figure 5:**
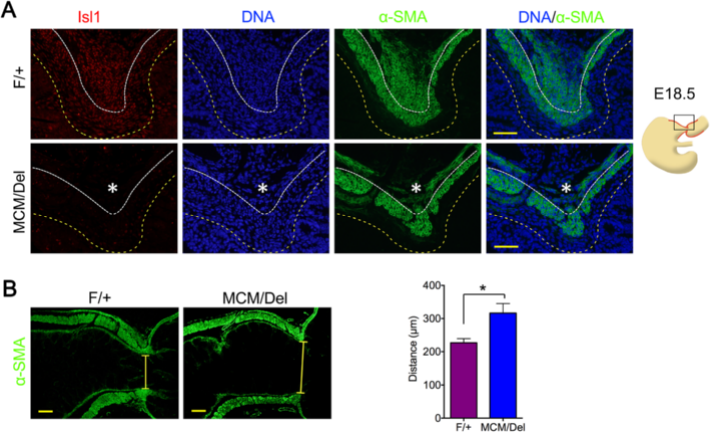
**Loss of Isl1 disrupts formation of the dorsal pyloric outer longitudinal muscle. (A)** Immunofluorescence of Isl1 and α-SMA in *Isl1*^*F/+a*^nd *Isl1*^*MCM/Del*^ embryonic pylorus at E18.5. Loss of Isl1 resulted in nearly complete loss of α-SMA-positive cells in the dorsal pyloric OLM (asterisks). Yellow dotted lines mark the epithelial basement membrane and white dotted lines indicate ICM and OLM boundary. Red staining is Isl1, green staining is α-SMA, and DAPI nuclear counterstaining (DNA) is blue. Scale bars: 50 μm. **(B)** α-SMA immunofluorescence of *Isl1*^*F/+a*^nd *Isl1*^*MCM/Del*^ embryonic pylorus at E18.5. Compared with *Isl1*^*F/+e*^mbryos, the pyloric sphincter constriction was wider in *Isl1*^*MCM/Del*^ animals. Sphincter constriction measurements are shown. Yellow bars demarcate the pylorus and highlight the marked difference in width between *Isl1*^*F/+a*^nd *Isl1*^*MCM/Del*^ samples. Data are mean ± SEM (*n* = 6 mice per group), *P <0.05 (Student’s *t*-test). Scale bars: 50 μm. α-SMA, α-smooth muscle actin; ICM, inner circular muscle; OLM, outer longitudinal muscle.

**Figure 6:**
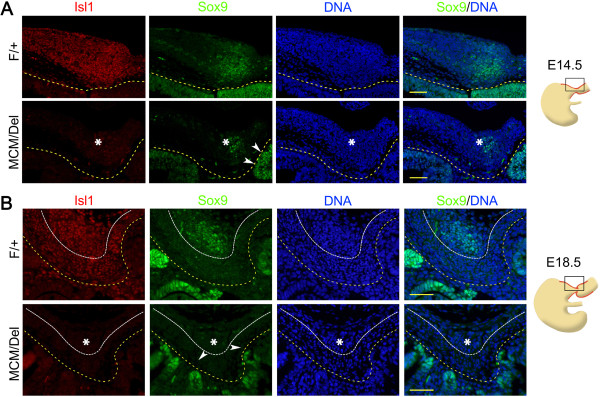
**Loss of Isl1 eliminates the dorsal pyloric outer longitudinal muscle Sox9 expression. (A)** Double immunostaining for Isl1 and Sox9 in the dorsal pylorus at E14.5. In the absence of Isl1, the domain of mesodermal cells (asterisks) expressing Sox9 was smaller. **(B)** Double immunostaining for Isl1 and Sox9 in the dorsal pylorus at E18.5. Inducible Isl1 knockout effectively eliminated Isl1 expression, with concomitant loss of Sox9 expression in the dorsal OLM cells (asterisks). Sox9 expression was normal in the stomach epithelium at both stages (arrowheads). Yellow dotted lines mark the epithelial basement membrane and white dotted lines separate ICM and OLM. Red staining is Isl1, green staining is Sox9, and DAPI nuclear counterstaining (DNA) is blue. Scale bars: 50 μm. ICM, inner circular muscle; OLM, outer longitudinal muscle.

To investigate effects of *Isl1* ablation on stomach development, we compared morphological and histological differences between *Isl1*^*MCM/Del*^ and *Isl1*^*F/+s*^tomachs at E18.5. At E18.5, yellow fluid was observed in *Isl1*^*MCM/Del*^ stomachs but not in stomachs of *Isl1*^*F/+l*^ittermates (Figure 4A, asterisk). Histological examination demonstrated that the dorsal pyloric smooth muscle layer was much thinner in the pylorus of *Isl1*^*MCM/Del*^ mice compared with controls (Figure 4B). We examined expression and distribution of α-SMA in both *Isl1*^*MCM/Del*^ mutants and *Isl1*^*F/+p*^ylorus. Immunofluorescence results demonstrated that Isl1 deficiency led to nearly complete absence of the pyloric OLM layer at E18.5, and remaining cells were loosely organized (Figure 5A, asterisks). In addition, constriction of the pyloric sphincter was attenuated in *Isl1*^*MCM/Del*^ mutant stomachs when compared with constriction in *Isl1*^*F/+s*^tomachs (Figure 5B). Furthermore, we analyzed expression of the smooth muscle specific protein Calponin-1 at E18.5, and immunofluorescence results demonstrated that loss of Isl1 also resulted in near absence of Calponin-1 expression in the dorsal pyloric OLM layer, similar to result with α-SMA (Additional file 1: Figure S5). Sox9 is expressed in both epithelium and mesenchyme [9] and is required for development of OLM and formation of pyloric sphincter constriction [20]. Our immunofluorescence results showed that Sox9 remained at normal levels in stomach epithelium of *Isl1*^*MCM/Del*^ mice at E14.5 and E18.5 (Figure 6, arrowheads), but the area of pyloric smooth muscle expressing Sox9 was significantly reduced in *Isl1*^*MCM/Del*^ mutants at E14.5 (Figure 6A, asterisks) and absent at E18.5 (Figure 6B, asterisks). Thus, *Isl1* was required for *Sox9* expression in dorsal pyloric OLM cells. These results indicate that Isl1 is critical for regulating development of mouse pyloric smooth muscle.

Expression and distribution of protein gene product 9.5 (PGP9.5), an enteric nervous system marker [32], was intact at E18.5 in *Isl1*^*MCM/Del*^ mutant stomachs (Additional file 1: Figure S6). Pancreatic and duodenal homeobox gene 1 (Pdx1) is expressed in epithelial cells of the antral-pyloric segment and the rostral duodenum [33]. Our immunofluorescence results showed that Pdx1 expression was similar in *Isl1*^*MCM/Del*^ mice when compared with controls at E18.5 (Additional file 1: Figure S7). In addition, the mouse stomach and duodenal epithelial boundary was established between E14.5 and E16.5 [34], this period coinciding with development of the OLM layer [20]. We tested the integrity of the stomach-small intestine epithelial pyloric border at E18.5 by examining expression of an intestine-specific epithelial marker Cdx2 [19]. Our immunohistochemistry results demonstrated that the position of the epithelial pyloric border in *Isl1*^*MCM/Del*^ mice was similar to that of controls (Additional file 1: Figure S8). These results indicate that loss of Isl1 does not affect innervation or epithelial development of the pylorus.

### Loss of Isl1 does not affect proliferation or apoptosis of pyloric inner circular muscle and outer longitudinal muscle cells

To see whether Isl1 expression was related to cell proliferation of the pylorus, we examined co-localization of Isl1 and the proliferative marker bromodeoxyuridine (BrdU) using immunofluorescence in *Isl1*^*F/+m*^ice. Our results showed that BrdU-positive cells were dense at E11.5 and scattered throughout the ICM and OLM regions at E14.5 and E18.5 (Additional file 1: Figure S9a). In addition, the proportion of proliferating ICM and OLM cells was not significantly different between *Isl1*^*MCM/Del*^ and *Isl1*^*F/+m*^ice at E18.5 (Additional file 1: Figure S9b).

To assess a potential impact on apoptosis, we examined cleaved Caspase 3 expression at E18.5, and our immunofluorescence results showed there were no Caspase 3-positive cells in pyloric ICM or the OLM layer of *Isl1*^*MCM/Del*^ and *Isl1*^*F/+m*^ice (Additional file 1: Figure S10). These data indicate that Isl1 ablation does not affect proliferation or apoptosis of pyloric ICM and OLM cells.

### Loss of Isl1 results in decreased expression of *Gata3*, *Gremlin*, and *Nkx2.5*

**Figure 7:**
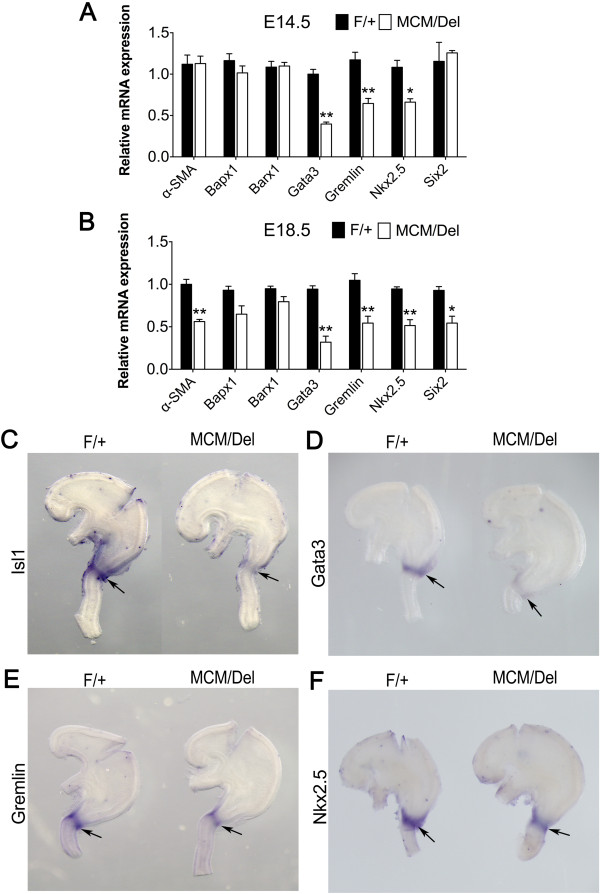
**Aberrant gene expression in hindstomach in *Isl1***^***MCM/Del ***^**mutants. (A)** RT-qPCR analysis of mRNA levels of hindstomach-enriched transcription factors at E14.5 indicates significant reduction of *α-SMA*, *Six2 Nkx2.5*, *Gata3*, and *Gremlin* mRNA in *Isl1*^*MCM/Del*^ mutant stomachs (*n* = 4). All results were normalized to levels of *Gapdh* mRNA. **(B)** RT-qPCR analysis of mRNA levels of hindstomach-enriched transcription factors at E18.5 indicates a significant reduction of *Nkx2.5*, *Gata3*, and *Gremlin* mRNA in the *Isl1*^*MCM/Del*^ mutant stomachs (*n* = 4). All results were normalized to levels of *Gapdh* mRNA. Data are mean ± SEM (n = *6* mice per group). *P <0.05 versus *Isl1*^*F/+*^; **P <0.01 versus *Isl1*^*F/+*^ (Student’s *t*-test). **(C-F)** WISH mRNA analysis confirmed loss of *Isl1*, *Gata3*, *Gremlin*, and *Nkx2.5* mRNA expression at E14.5 in the *Isl1*^*MCM/Del*^ mutant stomachs. *Isl1* and *Gata3* mRNA were severely down-regulated in *Isl1*^*MCM/Del*^ mice, whereas *Gremlin* and *Nkx2.5* expression were slightly reduced. Arrows point to the pyloric sphincter.

Since a numbers of factors, including *Bapx1*[18], *Barx1*[35], *Gata3*[19,36], *Gremlin*[5], *Nkx2.5*[2], and *Six2*[9], have been reported to be involved in regulation of pyloric development, we examined the effects of loss of Isl1 on expression of these genes at E14.5 and E18.5. RT-qPCR results showed that loss of *Isl1* did not affect expression of *Bapx1* or *Barx1* at either E14.5 or E18.5 (Figure 7A,B). At E18.5, *α-SMA* and *Six2* mRNA levels were significantly lower in *Isl1*^*MCM/Del*^ mice as compared with controls (Figure 7B). At both E14.5 and E18.5, *Nkx2.5*, *Gata3*, and *Gremlin* mRNA levels in the stomach of *Isl1*^*MCM/Del*^ mice were lower than controls (Figure 7A,B). *Gata3* mRNA levels were approximately 70% decreased at both stages examined (Figure 7A,B). Based on these results, we investigated *Isl1*, *Gata3*, *Gremlin*, and *Nkx2.5* expression in *Isl1*^*MCM/F*^ mutant and *Isl1*^*F/+s*^tomachs using WISH. Results demonstrated that expression of each of these genes was mainly confined to the pyloric region, as expected; *Gata3* expression was more reduced in mutant stomachs; and *Gremlin* and *Nkx2.5* only had subtle changes (Figure 7E,F). *Isl1* and *Gata3* expression were the most strongly affected (Figure 7C,D). These results were consistent with RT-qPCR data and suggest that Isl1 regulates expression of *Gata3*, *Gremlin*, and *Nkx2.5*.

### Isl1 targets *Gata3* and activates its transcription

**Figure 8:**
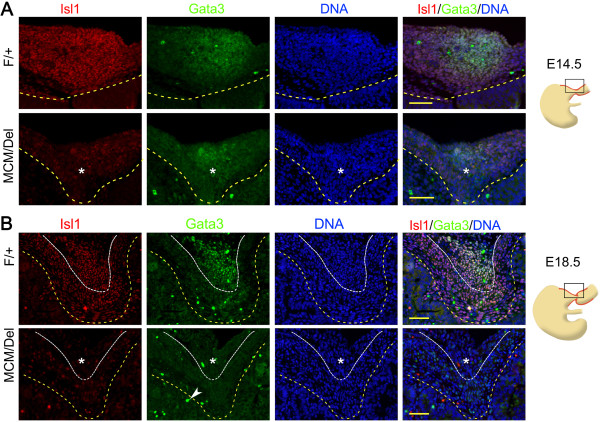
**Loss of Isl1 eliminates the dorsal pyloric outer longitudinal muscle Gata3 expression. (A)** Double immunostaining for Isl1 and Gata3 in the dorsal pylorus at E14.5. The region of mesodermal cells (asterisks) expressing Gata3 was smaller in the *Isl1*^*MCM/Del*^ pylorus than *Isl1*^*Fl+*^. **(B)** Double immunostaining for Isl1 and Gata3 in the dorsal pylorus at E18.5. Inducible Isl1 knockout effectively eliminated Isl1 expression, with concomitant loss of Gata3 expression in the dorsal OLM cells (asterisks). Yellow dotted lines mark the epithelial basement membrane and white dotted lines indicate the ICM and OLM boundary. White arrowhead indicates non-specific stain. Red staining is Isl1, green staining is Gata3, and DAPI nuclear counterstaining (DNA) is blue. Scale bars: 50 μm. ICM, inner circular muscle; OLM, outer longitudinal muscle.

**Figure 9:**
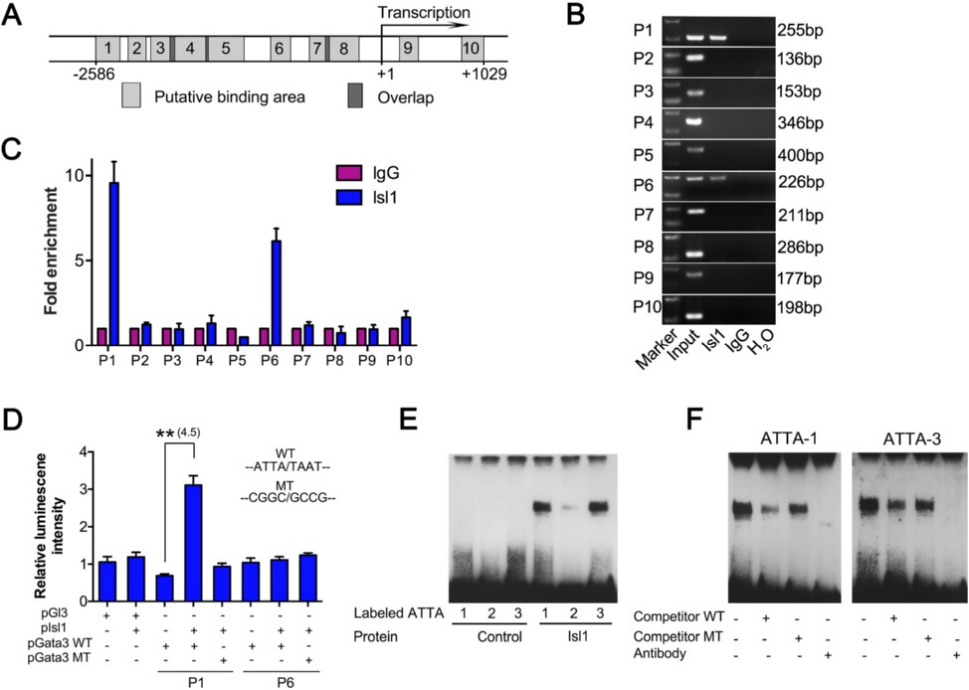
**Isl1 directly binds to *Gata3 *enhancer regions and regulates the *Gata3 *enhancer activity. (A)** A schematic representation of the *Gata3* gene surrounding the transcription start site. Putative Isl1 binding sequences (containing the ATTA/TAAT sequence) are shown as grey rectangles. **(B)** ChIP-PCR amplification was obtained using P1 to P10 primers which would amplify Isl1 consensus-containing fragments in the vicinity of the *Gata3* transcription start site. ChIP with Isl1 antibody and amplification of fragments using the indicated primers (Additional file 2: Table S3) demonstrated binding of Isl1 to the *Gata3* promoter regions in pylorus of wild-type mouse embryos at E14.5. A cell aliquot before precipitation was designated as the input sample. IgG was a negative control provided by the kit. **(C)** Fold change of enriched DNA fragment from ChIP detected by qPCR. **(D)** Effects of an Isl1 expression vector on the transiently transfected *Gata3* gene enhancers (P1 and P6 regions) fused to luciferase reporter genes in 293FT cells. Data are mean ± SEM (*n* = 4). **P <0.01 (Student’s *t*-test). **(E)** EMSA were performed with *in vitro* translated pcDNA3.1-Isl1 and control vector respectively. Isl1 efficiently bound to oligonucleotides representing number 1 and 3 sites of the Gata3-P1 enhancer region. **(F)** Labeled ATTA number 1 and 3 probes of the P1 region were incubated with *in vitro* translated pcDNA3.1-Isl1 protein and assayed by EMSA. Specificity of protein-DNA binding was determined by competition with excess unlabeled wild-type or mutant competitor oligonucleotides. Additionally, Isl1 binding to oligonucleotide probes was blocked by antibodies to Isl1. bp, base pairs; ChIP, chromatin immunoprecipitation; EMSA, electrophoretic mobility shift assays; IgG, immunoglobulin G; MT, mutant type; WT, wild type.

*Gata3* is selectively expressed in the pylorus of the developing mouse embryo [19,20]. Expression of both *Isl1* and *Gata3* mRNA was observed in the pylorus at E14.5, but whether Gata3 and Isl1 are expressed in the same cells has not been explored. Therefore, we examined expression of Isl1 and Gata3 by immunofluorescence analyses. Results demonstrated that Isl1 and Gata3 proteins were co-expressed within the same cells of the pylorus at E14.5 and E18.5 in *Isl1*^*F/+c*^ontrol stomachs (Figure 8). In addition, the area expressing Gata3 was significantly smaller in *Isl1*^*MCM/Del*^ mutant pyloric smooth muscle layer at E14.5 (Figure 8A) and it was lost at E18.5 in the pyloric OLM layer (Figure 8B). Thus, *Isl1* was required for *Gata3* expression in the dorsal pyloric OLM layer. To investigate whether Isl1 regulates pyloric development by directly regulating *Gata3*, we performed bioinformatics analysis of the Gata3 genomic locus. The mouse *Gata3* gene contains several putative Isl1 response elements (ATTA/TAAT) at -2,832 base pairs (bp) to +1,002 bp from the transcription initiation sites [37]. We identified 10 areas that contained a putative Isl1 binding site (Figure 9A), and 10 pairs of corresponding primers were designed to amplify these regions following chromatin immunoprecipitation (ChIP) studies utilizing antibody to Isl1. Immunoprecipitated genomic DNA was obtained from pyloric regions of mouse embryos at E14.5. Of the 10 putative Isl1 binding areas, two discrete regions, in the -2,558 bp to -2,303 bp (P1 region) and -1,081 bp to -855 bp (P6 region), were occupied by Isl1 protein. This result was confirmed by semi-quantitative PCR (Figure 9B) and the fold enrichment method (Figure 9C).

Luciferase assays were also performed to investigate the ability of Isl1 to regulate the Gata3-P1 or Gata3-P6 enhancer regions. Results of these luciferase reporter assays demonstrated that Isl1 overexpression enhanced activity of the Gata3-P1-wild-type luciferase reporter approximately 4.5-fold (Figure 9D). Site-directed mutagenesis revealed that mutation of the Isl1 consensus site within the P1 enhancer selectively decreased the ability of Isl1 co-transfection to activate the reporter. Isl1 expression did not affect luciferase activities of Gata3-P6-wild-type, Gata3-P6-mutant-type and pGL3.0-basic (Figure 9D). Together, the data strongly suggest that Isl1 regulates *Gata3* transcription by binding to the Gata3-P1 element at the -2,558 bp to -2,303 bp region.

To further investigate this, electrophoretic mobility shift assays (EMSA) were performed with *in vitro* translated pcDNA3.1-Isl1 and control vector respectively. The Gata3-P1 enhancer region included three putative ATTA binding sites, and Isl1 efficiently bound to oligonucleotides representing number 1 and 3 sites (Figure 9E). Binding of Isl1 to number 1 and 3 sites was specifically competed for by excess unlabeled probes but not by excess unlabeled probes containing mutations within the Isl1 consensus binding sites (Figure 9F). Additionally, binding to Isl1 consensus site containing oligonucleotides was blocked by Isl1 antibody. Collectively, these data demonstrate that Isl1 is a direct regulator of *Gata3* transcription.

## Discussion

The presented results show that Isl1 is highly expressed in early stages of stomach development in mouse embryos, being confined at later stages to the muscle layer of the pylorus. Previous results demonstrated that Isl1 expression in the developing stomach is restricted to the ventral gastric mesenchyme at E9.5 [29], and sharply increases until E13.5. During this period of time, the mouse stomach undergoes expansion from the foregut tube [9], and the circular muscle layer of the stomach forms [11]. Our results further demonstrate that Isl1 expression is localized to the posterior stomach mesenchyme from E11.5 to E13.5, and is concentrated in the smooth muscle cells of the pylorus at later stages of stomach development, although Isl1-positive cells are also detectable in the lamina propria. These results suggest that Isl1 might be involved in the regulation of stomach organogenesis and in development of the pyloric smooth muscle layer, which is derived from stomach mesenchyme. In support of this, ablation of Isl1 led to nearly complete absence of the pyloric OLM layer at E18.5.

Stomach organogenesis occurs after E9.5 during mouse development [9]. *Isl1* null mouse embryos show developmental anomalies at E9.5 and die at E10 [24]. To prolong the life of the embryos, we adopted a delayed knockout strategy using a tamoxifen-inducible mutated estrogen receptor ligand-binding domain (mER)-Cre-mER recombinase targeted to the *Isl1* locus, administering tamoxifen at E11.5. Our results are in agreement with a previous report that showed that the *Isl1*^*MCM/Del*^ mice died in the perinatal period [30]. We thus examined effects of *Isl1* ablation beginning at E18.5 on mouse stomach development during the subsequent embryonic development period. We found that Isl1 expression was effectively down-regulated at both gene and protein levels. Further morphological and histological results demonstrated that the dorsal pyloric smooth muscle layer was much thinner in the pylorus of *Isl1*^*MCM/Del*^ mice when compared with that of *Isl1*^*F/+m*^ice. Further evidence that Isl1 is required for formation and growth of the pylorus was that duodenogastric reflux, which results from reduced contractile activity of the pyloric sphincter [9,18], was clearly observed in *Isl1*^*MCM/Del*^ stomachs.

To investigate the cellular mechanisms by which loss of *Isl1* resulted in underdevelopment of the pylorus, we tested effects of *Isl1* ablation on pyloric cell differentiation, proliferation, and apoptosis. Loss of *Isl1* had no significant effects on pyloric cell proliferation or apoptosis. These results are consistent with previous results suggesting that Isl1 is not likely to be involved in promoting proliferation of gastrointestinal epithelium [29]. α-SMA is essential for muscle differentiation, and widely used as a smooth muscle marker [9]. The proportion of cells expressing α-SMA among Isl1-positive cells significantly increased from E11.5 to E18.5. *Isl1* ablation resulted in loss of the dorsal pyloric OLM layer and decreased *α-SMA* expression in *Isl1*^*MCM/Del*^ stomachs when compared to *Isl1*^*F/+a*^t E18.5. Therefore, we suggest that Isl1 affects pyloric development mainly by regulating dorsal pyloric OLM layer formation.

To reveal the molecular mechanisms by which Isl1 regulates pyloric development, we assessed the relationship between Isl1 and genes that are required for pyloric development, including *Bapx1*, *Barx1*, *Nkx2.5*, *Gremlin*, *Six2*, and *Gata3. Isl1*^*MCM/Del*^ mutants exhibited somewhat decreased expressions of *Nkx2.5* and *Gremlin*. Subtle changes in *Nkx2.5* and *Gremlin* expression may be owing to the loss of some muscle, where these genes were expressed. However, expression of *Gata3* was most dramatically down-regulated. Furthermore, *Gata3* deletion also abrogated development of the OLM layer, leading to loss of *Sox9* expression and pyloric constriction [20]. These results in *Gata3* null mice demonstrate that Gata3 is required for the survival of these smooth muscle cells, and stomachs are phenotypically similar to those observed in *Isl1*^*MCM/Del*^ mutants.

To investigate whether *Gata3* is a direct downstream target of Isl1 in stomach, we performed ChIP assays utilizing Isl1 antibody and chromatin from embryonic stomach, and EMSA assays with *in vitro* translated Isl1 protein. We found direct binding of Isl1 to several consensus Isl1 response elements in regions surrounding the *Gata3* transcription start site. In addition, co-transfection studies demonstrated the ability of an Isl1 expression vector to activate expression of the defined *Gata3* enhancer element. Collectively, our data demonstrate that Isl1 directly interacts with enhancer elements in the *Gata3* promoter region in stomach to activate Gata3 expression at the transcriptional level.

**Figure 10:**
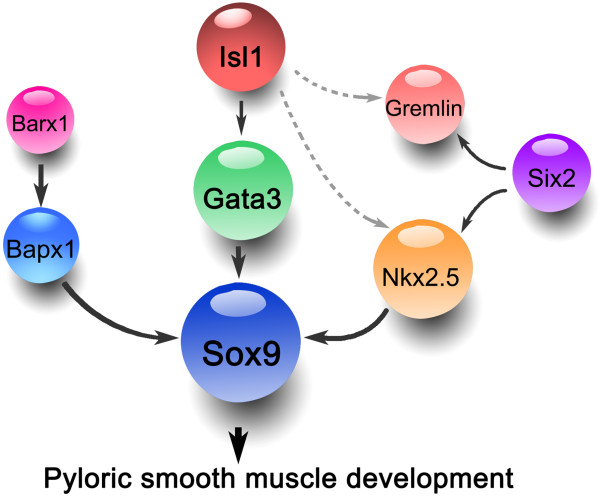
**Model of Isl1 function in mouse developing pyloric muscle. ***Bapx1* is lost in *Barx1*-null stomachs, *Barx1* functions upstream of *Bapx1*, and loss of *Bapx1* down-regulates *Sox9* expression. We therefore suggest that *Barx1* regulates *Sox9* through *Bapx1*. Loss of *Six2* reduces *Nkx2.5*, *Gremlin*, and *Sox9* expression, and loss of *Nkx2.5* also leads to loss of *Sox9* expression. In addition, *Sox9* is absent after deletion of *Gata3*. Our results demonstrate that Isl1 directly regulates *Gata3*, which suggests that *Sox9* is regulated by Isl1 via *Gata3*. Dotted lines indicate that *Nkx2.5* and *Gremlin* are down-regulated in *Isl1*^*MCM/Del*^ stomachs, but specific regulatory mechanisms still remain unclear.

Based on results presented here and previously published for mouse pyloric development, we propose a model for a molecular interaction network controlling pyloric development (Figure 10). *Bapx1* expression is lost in *Barx1*-null stomachs, and loss of *Bapx1* does not affect *Nkx2.5* expression, but gene expression microarrays show decreased Sox9 [18,38]. Thus, *Barx1* may regulate *Sox9* through *Bapx1*. Loss of *Six2* reduces *Nkx2.5*, *Gremlin*, and *Sox9* expression in pylorus [9], and *Nkx2.5* null stomachs also lead to loss of *Sox9* expression [20]; so, it is possible that *Sox9* is regulated by *Six2* through *Nkx2.5.* In addition, *Sox9* is absent after deletion of *Gata3*, and there is no direct relationship between *Gata3* and *Nkx2.5*[20], and our results demonstrate that Isl1 directly regulates *Gata3*, which suggests that *Sox9* is regulated by Isl1 via *Gata3*. Thus, all of these pathways converge on *Sox9* and confirm the critical role of *Sox9* in pyloric development.

Our study demonstrated that Isl1 is highly expressed in the developing mouse stomach and in particular in the pylorus. Functionally, Isl1 is required for pyloric OLM layer development. We have further shown that Isl1 directly targets *Gata3*. Reduced expression of Gata3 can account for the pyloric phenotype observed in Isl1 mutants. In light of the results presented here, Isl1 is critical for stomach organogenesis and pyloric OLM development. These findings are important for our understanding of diseases resulting from abnormalities of pyloric sphincter development.

## Conclusions

This work sheds new light on Isl1 expression and gives mechanistic insight into *Isl1* function in developing pylorus of mouse embryos. We found that Isl1 was strongly expressed in the posterior stomach of mouse embryos and mainly confined to the muscle layer of the pylorus. In addition, the proportion of Isl1-positive cells expressing α-SMA gradually increased in the pylorus as development progressed and loss of *Isl1* resulted in loss of the dorsal pyloric OLM layer in *Isl1*^*MCM/Del*^ stomachs at E18.5. These new findings demonstrate that Isl1 is involved in regulating pyloric OLM development. Subsequent analysis further revealed that Isl1 ensures normal stomach pyloric development via directly targeting *Gata3*. These findings are highly clinically relevant and will help us to better understand the cause of related diseases such as hypertrophic pyloric stenosis resulting from smooth muscle hypertrophy in the pylorus.

## Methods

### Animals

Adult (6- to 8-week-old) male and female C57BL/6 mice were used for this study. All animal studies were approved by the Chinese Association for Laboratory Animal Sciences. The age of mouse embryos was determined by the appearance of the vaginal plug, which was taken to be E0.5. The birth day of the pup was marked as P1 for these experiments. Generations of *Isl1*^*MCM/+a*^nd *Isl1*^*F/F*^ mice have been reported previously [30,31]. In brief, we used a ‘floxed’ *Isl1* allele (*Isl1*^*F*^) in which *loxP* sites were inserted into the introns flanking exon 4 of the *Isl1* locus [30], and a tamoxifen-inducible knockin Isl1 mER-Cre-mER allele [31,39]. *Isl1*^*F/F*^ mice were mated with *Isl1*^*MCM/+m*^ice to generate litters with equal numbers of *Isl1*^*MCM/F*^-inducible knockouts (*Isl1*^*MCM/Del*^) and *Isl1*^*F/+c*^ontrols. To induce excision in *Isl1*^*MCM/F*^ embryos, pregnant females were administered an oral gavage of 300 μl of tamoxifen (T5648; Sigma, St. Louis, MO, USA) in sesame oil (10 mg/ml) at E11.5 for three consecutive days just before Isl1 expression sharply increased, and the embryos were harvested at E14.5 or E18.5.

### Patient material

Two patients with hypertrophic pyloric stenosis were selected from the 306th Hospital of People’s Liberation Army, Beijing. Pyloric tissue stored in the 4% Paraformaldehyde buffered in 0.01M PBS were selected from excess material collected from patients undergoing operations to retrieve surgical specimens. The study on human material was performed according to the instructions and guidelines of the 306th Hospital Ethics Committee. Approval of this study was granted by the Chinese Association for Laboratory Animal Sciences and the 306th Hospital Ethics Committee.

### PCR, semi-quantitative PCR and real-time quantitative PCR

Genomic DNA was isolated from tail biopsies following the HotSHOT method [40] and genotyping was performed using standard PCR methods with sequence-specific primers (Additional file 2: Table S1). Total RNA was extracted from the pyloric regions of stomachs at E14.5 and E18.5 using commercial reagents (12183–016; Invitrogen, Carlsbad, CA, USA), according to the manufacturer’s instructions. RNA was converted to cDNA using M-MLV reverse transcription reagents (M170A; Promega, Madison, WI, USA). RT-qPCR was performed using SYBR Green master mix (DRR420A; TaKaRa, Dalian, China) in the ABI PRISM 7500 Sequence Detection System (Applied Biosystems, Foster City, CA, USA) and reactions were done in triplicate. RT-qPCR conditions were as follows: 95°C for 2 minutes, followed by 40 cycles of 95°C for 15 seconds and 60°C for 1 minute. Relative RNA quantifications were normalized to endogenous control *Gapdh*. PCR and semi-quantitative PCR was performed in the PCR instrument (Bio-Rad Laboratories, Hercules, CA, USA) as follows: 94°C for 5 minutes (one cycle); 94°C for 30 seconds, 60°C for 30 seconds, 72°C for 30 seconds (32 cycles); 72°C for 10 minutes; and 4°C holding. PCR products were visualized on a 2% agarose gel with added ethidium bromide. Primers for detecting Isl1 knockdown efficiency and identifying gene expression change in *Isl1*^*MCM/Del*^ mouse embryos are listed in Additional file 2: Table S1.

### Western blot

Embryonic stomachs were lysed with RIPA buffer (9806; Cell Signaling, Danvers, MA, USA) containing 1 mM phenylmethylsulfonyl fluoride (8553S; Cell Signaling). The protein concentration of each group was determined using a bicinchoninic acid assay reagent (Vigorous Biotechnology, Beijing, China) according to the manufacturer’s recommendations. Equal amount of proteins were electrophoresed on a 12% SDS-PAGE, and the bands were transferred onto polyvinylidene difluoride (PVDF) membranes (Bio-Rad Laboratories). The membrane was blocked with 5% (w/v) non-fat dry milk for 3 hours and incubated with Isl1 antibody (40.2D6; Developmental Studies Hybridoma Bank, Iowa City, IA, USA) and internal control Gapdh antibody (AM4300;= Ambion, Austin, TX, USA) overnight at 4°C. The PVDF membrane was then washed three times for 30 minutes in 0.1% Tween-20 in Tris-buffered saline (TBST) and incubated for 1 hour with horseradish peroxidase-conjugated goat anti-mouse IgG (Zhongshan, Beijing, China). After washing for 30 minutes with three changes of TBST, the membrane was treated with the pierce™ ECL 2 Western Blot Substrate (Thermo Scientific, Rockford, IL, USA).

### Chromatin immunoprecipitation

Chromatin was prepared from the pyloric regions of C57BL/6 mouse embryos at E14.5 using the manufacturer’s instructions (17–371; Millipore, Darmstadt, Germany). Tissues were dissected in ice-cold PBS. Following a gentle digestion, cells were cross-linked with 1% formaldehyde (252549, Sigma) and chromatin was sheared by sonication to an average length of 500 bp. The antibody used for immunoprecipitation was the 39.4D5 Isl1 (Developmental Studies Hybridoma Bank). Reverse cross-linked immunoprecipitated chromatin was subjected to both RT-PCR and RT-qPCR using primers corresponding to the specific region (spanning the 10 putative Isl1 binding sites). Primers are listed in Additional file 2: Table S3. In all, we collected pylorus regions of more than 400 embryos and repeated ChIP reactions four times.

### Plasmid construction

P1 and P6 regions of *Gata3* and *α-SMA*[41] promoter gene were amplified from mouse genomic DNA by RT-PCR method using specific primers. Primers are listed in Additional file 2: Table S2. The forward primer contained a restriction enzyme site of *SacI* and the reverse primer contained a restriction enzyme site of *HindIII*. The PCR product was purified from agarose gel, digested, and cloned into *SacI* and *HindIII* sites of pGL3.0-basic luciferase reporter vector (E1910; Promega). Mutated Gate3-P1 and Gata3-P6 promoter regions were done using over-lap PCR, and ATTA/TAAT were mutated to CGGC/GCCG in each putative Isl1 binding site sequence. All of the constructs were verified by sequencing.

### Transient transfection and luciferase assays

Human embryonic cells (293FT) were cultured in Dulbecco’s modified Eagle’s medium with 10% fetal bovine serum (Invitrogen) supplemented with 100 IU/ml penicillin and 100 IU/ml streptomycin. 293FT cells were plated at a density 5 × 10^4^ cells per well in 24-well plates. After 24 hours in culture, cells were transfected with the Isl1 expression vector (Institute of Molecular and Cell Biology, Singapore) or pXJ40-Myc control vector, *Gata3* or *α-SMA* luciferase reporter vectors, and pTK-Ranilla vector (E2241; Promega) at a ratio of 10:4:1 using the VigoFect transfection reagent (Vigorous Biotechnology). Cells were harvested 24 hours after transfection. Using the same method, the pcDNA-Gata3 expression vector (plasmid 1332; Addgene, Cambridge, MA, USA) and *α-SMA* luciferase reporter vector were co-transfected into 293FT cells. Luciferase activity was measured using a dual-luc assay kit (E1960; Promega) on a Modulus™ Microplate Luminometer (Turner Biosystems, Sunnyvale, CA, USA). Values shown by the fluc to rluc ratio were normalized to an empty luciferase reporter control. All transfection experiments were performed at least three times.

### Hematoxylin and eosin staining

Hematoxylin and eosin staining was performed as previously described [42]. Briefly, sections were dewaxed, rehydrated, stained with hematoxylin, incubated in bluing solution, counterstained with eosin, dehydrated, and equilibrated with xylene. Glass coverslips were mounted with Permount Mounting Media (SP15-100; Fisher Scientific, Pittsburgh, PA, USA). Sections were photographed under bright-field microscope photograph system (Leica Microsystems, Buffalo Grove, IL, USA).

### Immunofluorescence

Stomach samples or embryos were fixed in 4% paraformaldehyde in PBS and embedded in paraffin. Serial sections were dewaxed and rehydrated, and antigen retrieval was performed by microwaving the sections in 0.01 M sodium citrate buffer (pH 6.0). Sections were then blocked using 10% normal animal serum in PBS for 1 hour at room temperature, and incubated with primary antibodies overnight at 4°C. Subsequently, sections were washed and incubated with appropriate secondary antibodies for 2 hours at room temperature. For signal amplification, slides were washed and incubated with appropriate tertiary antibodies for 2 hours. Sections were counterstained with DAPI (10236276001; Roche Applied Science, Basel, Switzerland) for 10 minutes and mounted on plus-coated slides that were cover-slipped using Vectashield (H-1000; Vector Laboratories, Burlingame, CA, USA). Finally, sections were photographed under a fluorescence microscope photograph system (Leica Microsystems).

Primary antibodies used were goat polyclonal to Isl1 (AF1837; R&D, Minneapolis, MN, USA); mouse monoclonal to α-SMA (A2547; Sigma); mouse monoclonal to Gata3 (sc-268; Santa Cruz Biotechnology, Santa Cruz, CA, USA); rabbit polyclonal to Pdx1 (ab47267; Abcam, Cambridge, UK); rabbit polyclonal to PGP9.5 (AB1761; Millipore); rabbit polyclonal to Sox9 (AB5535; Millipore); rabbit monoclonal to cleaved Caspase 3 (9664S; Cell Signaling), and mouse polyclonal to BrdU (G3G4; Developmental Studies Hybridoma Bank). Secondary antibodies used were biotinylated conjugated donkey anti-goat IgG (sc-2042; Santa Cruz Biotechnology), CY2-conjugated goat anti-mouse IgG (115-225-146; Jackson ImmunoResearch, West Grove, PA, USA), and 488 donkey anti-rabbit IgG (A21206; Life Technologies, Carlsbad, CA, USA). Tertiary antibodies used were TRITC-conjugated streptavidin (7100–03; SouthernBiotech, Birmingham, AL, USA). See Additional file 2: Table S4 for details of specific immunofluorescence protocols.

For BrdU immunofluorescence, DNA was denatured in 2 N HCl at 37°C for 30 minutes and BrdU-incorporated sites were exposed by 0.01% trypsin at 37°C for 12 minutes. After incubation with animal serum, other-step process described above.

### Immunohistochemistry

Paraffin sections were processed as described above (see Immunofluorescence). Mouse monoclonal antibody to Cxd2 (AM392; BioGenex, San Ramon, CA, USA) and Isl1 antibody (40.2D6; Developmental Studies Hybridoma Bank, Iowa City, IA,USA) were incubated on sections overnight at 4°C. Sections were washed and incubated with a biotinylated goat anti-mouse IgG (115-065-146; Jackson ImmunoResearch) for 2 hours at room temperature. Slides were then washed and incubated for horseradish peroxidase-conjugated streptavidin (123-065-021; Jackson ImmunoResearch) for 2 hours at room temperature. Peroxidase activity was detected with the addition of diaminobenzidine (D4293; Sigma) and 0.1% H_2_O_2_. Sections were counterstained with hematoxylin, dehydrated, and covered with coverslips. Sections were photographed as described above (see Hematoxylin and eosin staining). See Additional file 2: Table S4 for details of specific immunohistochemistry protocols.

### Measurement of pyloric sphincter constriction

A single section from at least six independent I*sl1*^*F/+a*^nd *Isl1*^*MCM/Del*^ embryos was examined by immunofluorescence for α-SMA, as described above (see Immunofluorescence). The shortest distance between the smooth muscle layers on opposite sides of the pyloric lumen was measured with Image J (United States National Institutes of Health, Bethesda, MA, USA) [43].

### BrdU labeling

BrdU was conducted by intraperitoneal injection of BrdU (50 mg/kg) into the pregnant female 2 hours before euthanasia by cervical dislocation. The embryos were removed and analyzed as described above.

### Whole mount *in situ* hybridization

WISH was performed as previously described [44]. Tissues were fixed in 4% paraformaldehyde for 4 hours, dehydrated in methanol, and stored in 100% MeOH at -20°C until use. Samples were rehydrated, pretreated with proteinase K, and hybridized with DIG-labeled cRNA probes after washing with 2× SSC/50% formamide three times at 70°C. The signal was detected using an alkaline phosphatase-conjugated anti-DIG antibody (11093274910; Roche). Tissues were incubated in the BM Purple alkaline phosphatase substrate (11442074001; Roche) at 4°C for several hours until the signal developed to the desired extent. Probes for *Gata3* 564 nucleotide (1028 to 1591 bp), *Nkx2.5* 825 nucleotide (628 to 1452 bp), *Gremlin* 550 nucleotide (758 to 1307 bp), and *Isl1* 780 nucleotide (524 to 303 bp) were generated using DIG RNA Labeling Kit (11 175 025 910; Roche). Primers are provided in Additional file 2: Table S2.

### Electrophoretic mobility shift assays

pcDNA3.1-Isl1 plasmid was used as a template for *in vitro* transcription and translation of Isl1 using the TNT Coupled Reticulocyte Lysate System (Promega; L4611) and pcDNA3.1 was used as control. 5′-biotin-labeled oligonucleotides were obtained from Sangon Biotech (Shanghai, China). Double-stranded DNA probes were generated by incubating complementary oligonucleotides at 90°C for 5 minutes, room temperature for 15 minutes, and 4°C for 5 minutes in a buffer containing 10 mM Tris, 1 mM EDTA and 100 mM NaCl (pH 8.0). pcDNA3.1-Isl1 was generated by cloning a fragment encoding C-terminal 216 amino acids of Isl1 into the pcDNA3.1/Hygro (+) vector. N-terminal 133 amino acids including Isl1 LIM domains have been shown previously to inhibit DNA binding *in vitro*[45]. Recombinant Isl1 protein was prepared by pcDNA3.1-Isl1 *in vitro* transcription and translation using the TNT Coupled Reticulocyte Lysate System (L4611; Promega) and pcDNA3.1 was used as control. DNA binding reactions (20 μl final volume) were proceeded at room temperature for 20 minutes in 1 × binding buffer (40 mM KCl, 15 mM HEPES (pH 7.9), 1 mM EDTA, 0.5 mM DTT, 5% glycerol and 50 ng/μl poly (dI•dC)) containing 2 μl of *in vitro* translated recombinant Isl1 or control reticulocyte lysate and 2 nM of 5′-biotin-labeled oligo probe. Oligonucleotide sequences were as follows: number 1 wild type: GTCCTCTTTCCCAATTACCCACTGTCAGTC, mutant: GTCCTCTTTCCCACGGCCCCACTGTCAGTC; number 2 wild type: GGACCGGCTGGGAATTACATGTTAAATACC, mutant: GGACCGGCTGGGACGGCCATGTTAAATACC; number 3 wild type: CCTGGAGGGGCCTATTAGATATTTTGTTTT, mutant: CCTGGAGGGGCCTCGGCGATATTTTGTTTT. Competition experiments were performed using 100-fold excess of unlabeled wild-type or mutant oligonucleotides pre-incubated with the Isl1 protein at room temperature for 10 minutes before adding the DNA probes. Antibody super-shift assays were performed using 1 μl of Isl1 antibody (40.2D6, 400 μg/mL) pre-incubated with Isl1 protein at room temperature for 20 minutes before adding the DNA probes. All DNA binding samples were electrophoresed on 6% non-denaturing polyacrylamide gels at 100 V for 45 minutes in 0.5 × tris-borate-EDTA buffer. Gels were transferred to a nylon membrane at 380 mA for 45 minutes in 0.5 × tris-borate-EDTA buffer. The biotin-labeled DNA was detected with a LightShift chemiluminescent EMSA kit (20148; Thermo Scientific).

### Statistical analysis

Data are expressed as means ± SEM. Statistical analysis was performed with GraphPad Prism 6.0. Comparisons between two groups were analyzed by Student’s *t*-test. More than two groups were compared using a one-way factorial analysis of variance, followed by Student’s *t*-test. A value of P <0.05 was considered to be statistically significant.

## Abbreviations

α-SMA: α-smooth muscle actin; bp: base pair; BrdU: bromodeoxyuridine; ChIP: chromatin immunoprecipitation; E: embryonic day; EMSA: electrophoretic mobility shift assays; Gata3: GATA binding protein 3; ICM: inner circular muscle; IgG: immunoglobulin G; Isl1: Insulin gene enhancer protein; Isl1F/F: *Isl1*^*flox/flox*^; Isl1MCM/Del: *Isl1*^*MCM/F*^-inducible knockout; LIM-HD: LIM homeodomain; mER: mutated estrogen receptor ligand-binding domain; OLM: outer longitudinal muscle; PBS: phosphate-buffered saline; Pdx1: Pancreatic and duodenal homeobox 1; PGP9.5: Protein gene protein 9.5; PVDF: polyvinylidene difluoride; RT-qPCR: real-time quantitative PCR; TBST: Tween-20 in Tris-buffered saline; WISH: whole mount *in situ* hybridization.

## Competing interests

The authors declare that they have no competing interests.

## Authors’ contributions

YSL and JRP were involved in experiment design, acquisition of data, analysis and interpretation of data, and drafting of the manuscript. CW, JC, YL, JLL, and XXZ performed experiments. SME was involved in critical revision of the manuscript for important intellectual content and provided *Isl1*^*F/F*^ and *Isl1*^*MCM/+m*^ice. YC was involved in study concept and design, critical revision of the manuscript for important intellectual content, and technical support, and provided human material. SC was involved in study concept and design, critical revision of the manuscript for important intellectual content, obtaining funding, and study supervision. All authors read and approved the final manuscript.

## Supplementary Material

Additional file 1 **Supplementary Information.** This file contains Figures S1 to S10. Click here for file

Additional file 2 **Supplementary Information.** This file contains Tables S1 to S4. Click here for file
