# Supplementary material for: LIM homeodomain transcription factor Isl1 directs normal pyloric development by targeting Gata3
Source: BMC Biol. 2014 Mar 27;12:25. doi: 10.1186/1741-7007-12-25 (PMC4021819; doi:10.1186/1741-7007-12-25)
Supplement: Additional file 1 — Supplementary Information. This file contains Figures S1 to S10. [file 1741-7007-12-25-S1.doc]

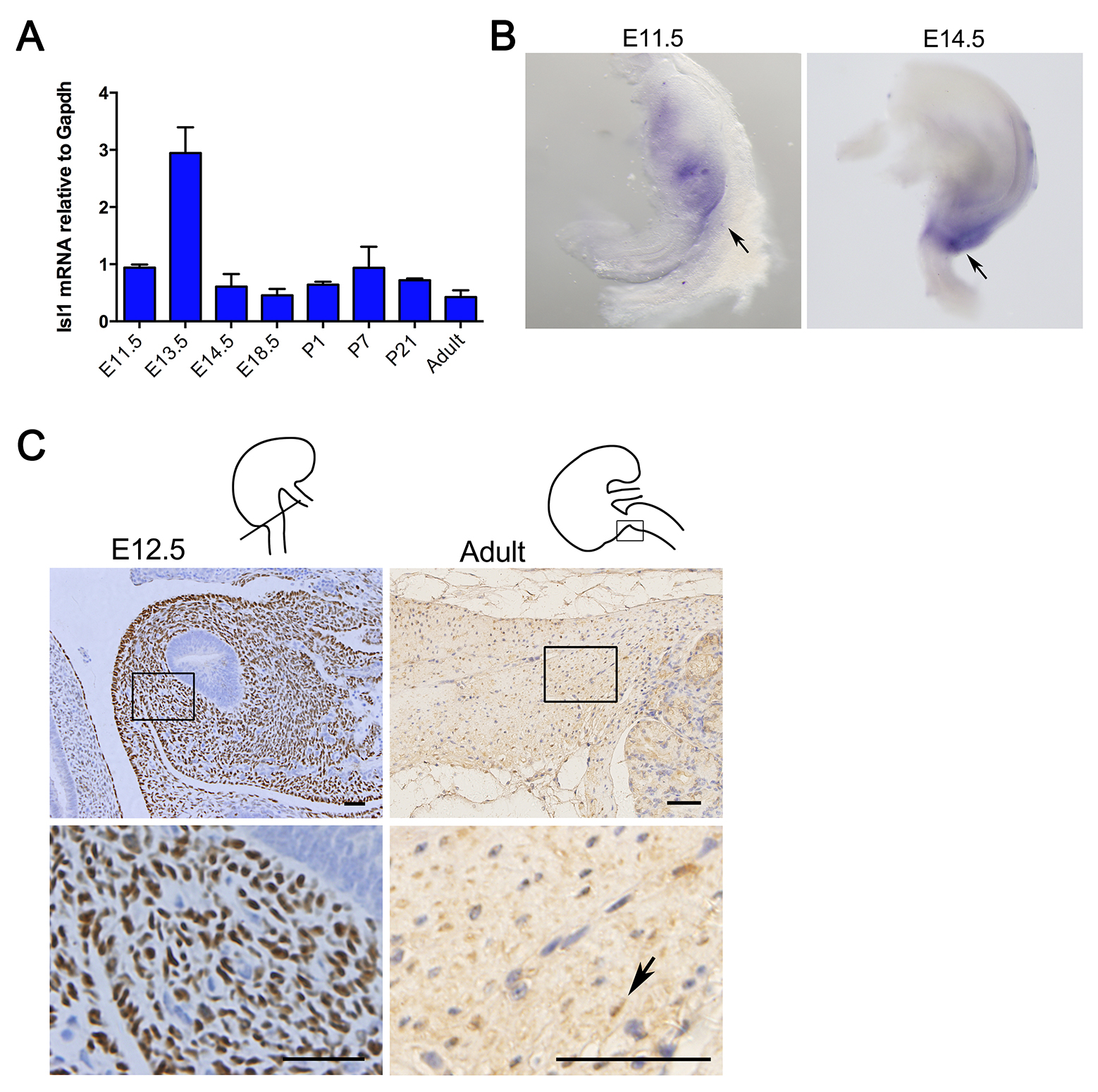


**Figure S1: Isl1 is expressed in developing mouse stomach.** (**a**) Relative mRNA levels of *Isl1* at different stages of stomach development were measured by RT-qPCR (n = 4). All results were normalized to levels of *Gapdh* mRNA. (**b)** At E11.5, Isl1 was expressed in the posterior stomach (arrow). By E14.5, Isl1 expression was limited to the pylorus (arrow). (**c**) Immunohistochemical staining for Isl1 in the stomach. Isl1 expression was mainly located in the mesenchyme of the posterior stomach at E12.5. In adult, Isl1 expression remained in the pylorus. Arrow shows representative Isl1 positive cell. Enlarged images of the boxed regions are shown below the original images. Scale bars of original images: 100 μm; Scale bars of enlarged images: 50 μm.


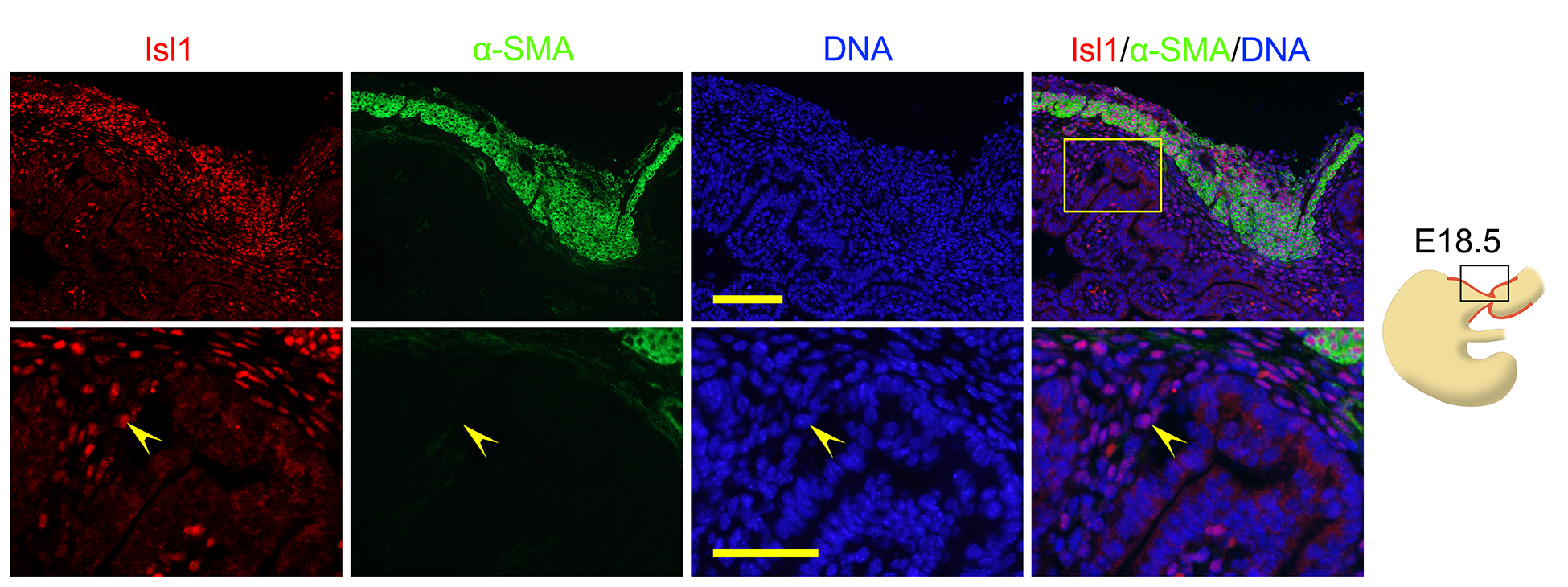


**Figure S2: Double immunostaining for Isl1 and α-SMA in lamina propria cells of the pylorus.** Isl1 was expressed in lamina propria cells at E18.5. Enlarged images in boxed regions are shown below original photos. Arrowheads denote representative Isl1 positive cells. Red staining is Isl1, green staining is α-SMA, and DAPI nuclear counterstaining (DNA) is blue. Scale bars: 50 μm.


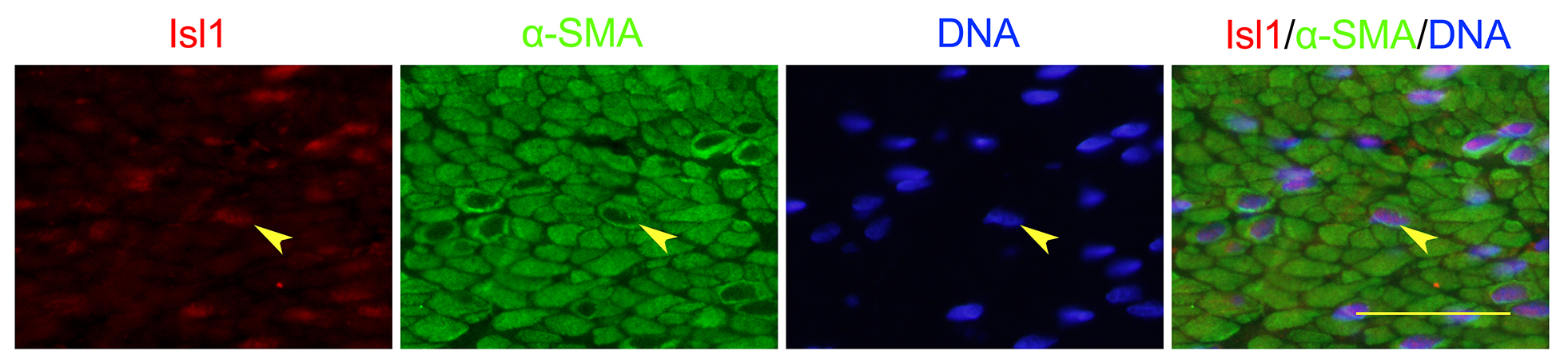


**Figure S3: Double immunostaining for Isl1 and α-SMA in human pylorus samples from patients with hypertrophic pyloric stenosis.** Red staining is Isl1, green staining is α-SMA, and DAPI nuclei counterstaining (DNA) is blue. Arrowheads show representative positive cell. Scale bars: 25 μm.


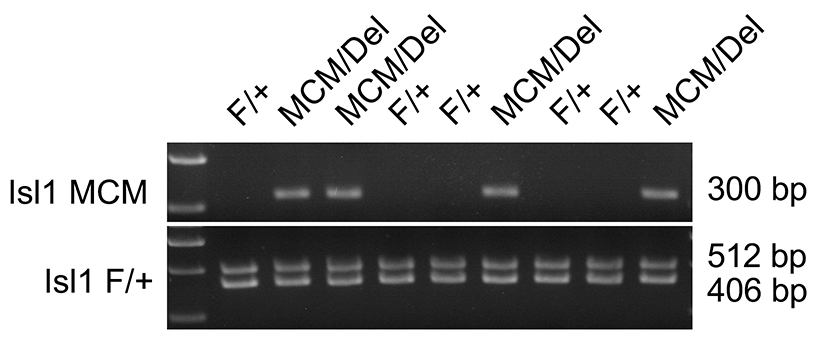


**Figure S4: Genotyping of inducible knockouts.** Genotyped was performed using PCR methods with sequence–specific primers. *Isl1^F/+^* alleles showed a 512 bp and a 406 bp PCR product. *Isl1^MCM/Del^* mice generated a 300 bp product while control mice didn’t generate any band.


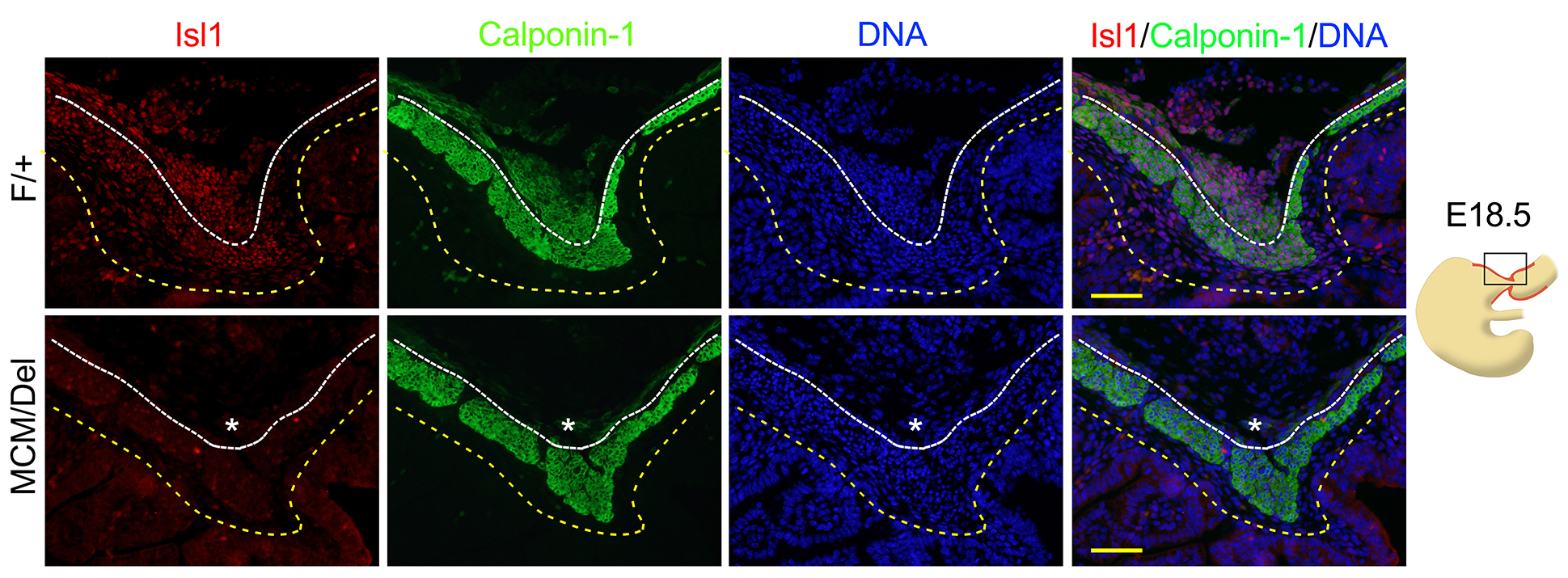


**Figure S5: Immunofluorescence of Isl1 and Calponin-1 in *Isl1^F/+^* and *Isl1^MCM/Del^* embryonic pylorus at E18.5.** Deficiency of Isl1 resulted in nearly complete loss of Calponin-1 positive cells in the dorsal pyloric outer longitudinal muscle (asterisks). Yellow dotted lines mark the epithelial basement membrane and white dotted lines indicate ICM and OLM boundary. Red staining is Isl1, green staining is Calponin-1, and DAPI nuclear counterstaining (DNA) is blue. Scale bars: 50 μm.


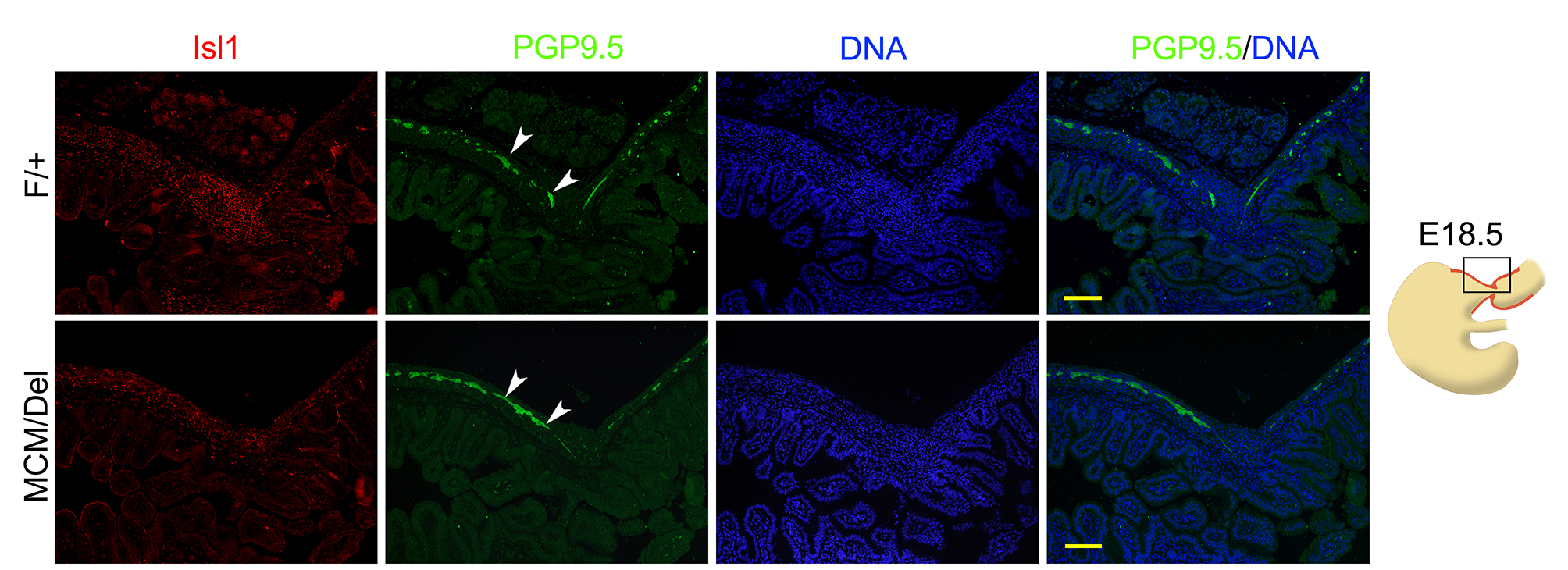


**Figure S6: Immunofluorescence of Isl1 and PGP9.5 in *Isl1^F/+^* and *Isl1^MCM/Del^* dorsal pylorus at E18.5.** Loss of Isl1 does not affect expression and distribution of PGP9.5 protein (arrowheads). Red staining is Isl1, green staining is PGP9.5, and DAPI nuclear counterstaining (DNA) is blue. Scale bars: 100 μm.


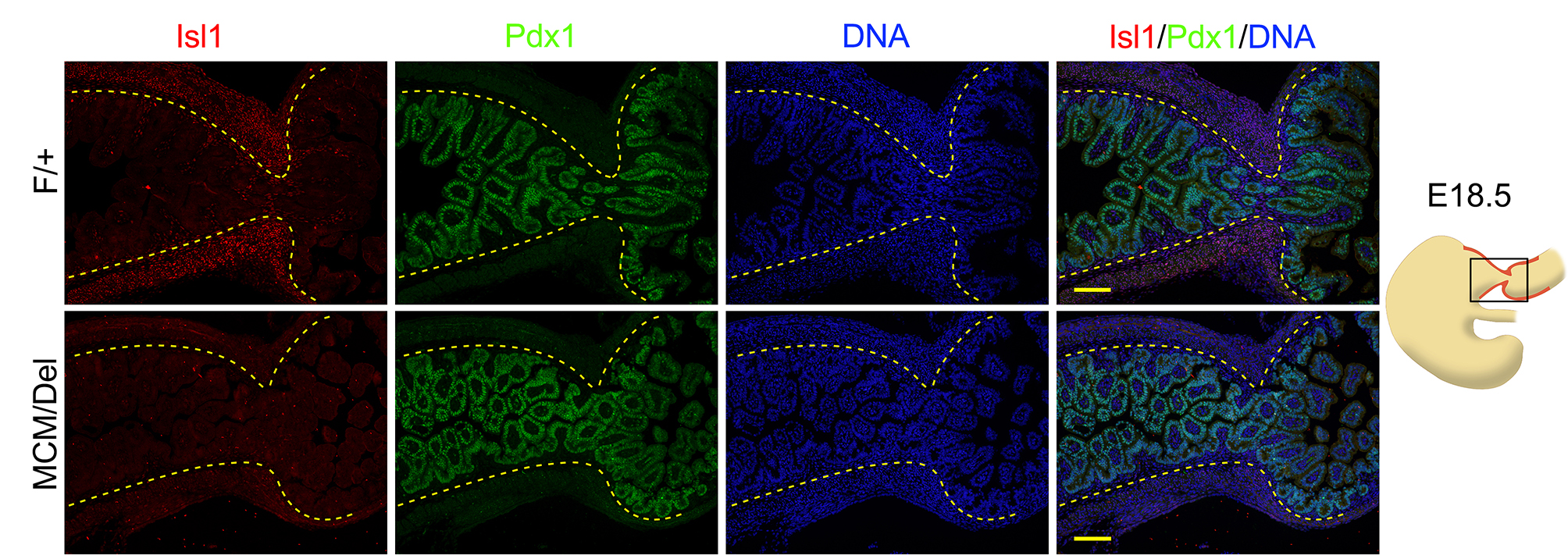


**Figure S7: Immunofluorescence of Isl1 and Pdx1 in *Isl1^F/+^* and *Isl1^MCM/Del^* pylorus at E18.5.** Pdx1 expression also is similar in *Isl1^MCM/Del^* mutant stomach epithelial cells when compared to *Isl1^F/+^* stomachs. Yellow dotted lines mark the epithelial basement membrane. Red staining is Isl1, green staining is Pdx1, and DAPI nuclear counterstaining (DNA) is blue. Scale bars: 50 μm.


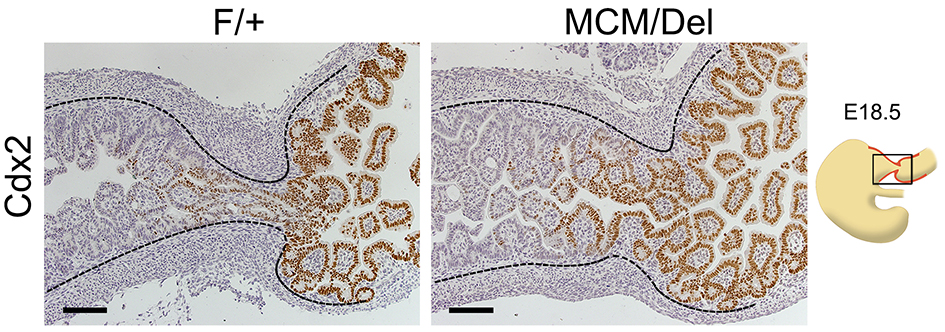


**Figure S8: The epithelial pyloric border is unaltered in the absence of Isl1.** Cdx2 immunohistochemistry in *Isl1^F/+^* and *Isl1^MCM/Del^* mice at E18.5. In *Isl1^MCM/Del^* mutants, despite concomitant pyloric muscular hypoplasia, the boundary of epithelial Cdx2 expression was preserved when compared to that in *Isl1^F/+^* littermates. Black dotted lines mark the epithelial basement membrane. Scale bars: 50 μm


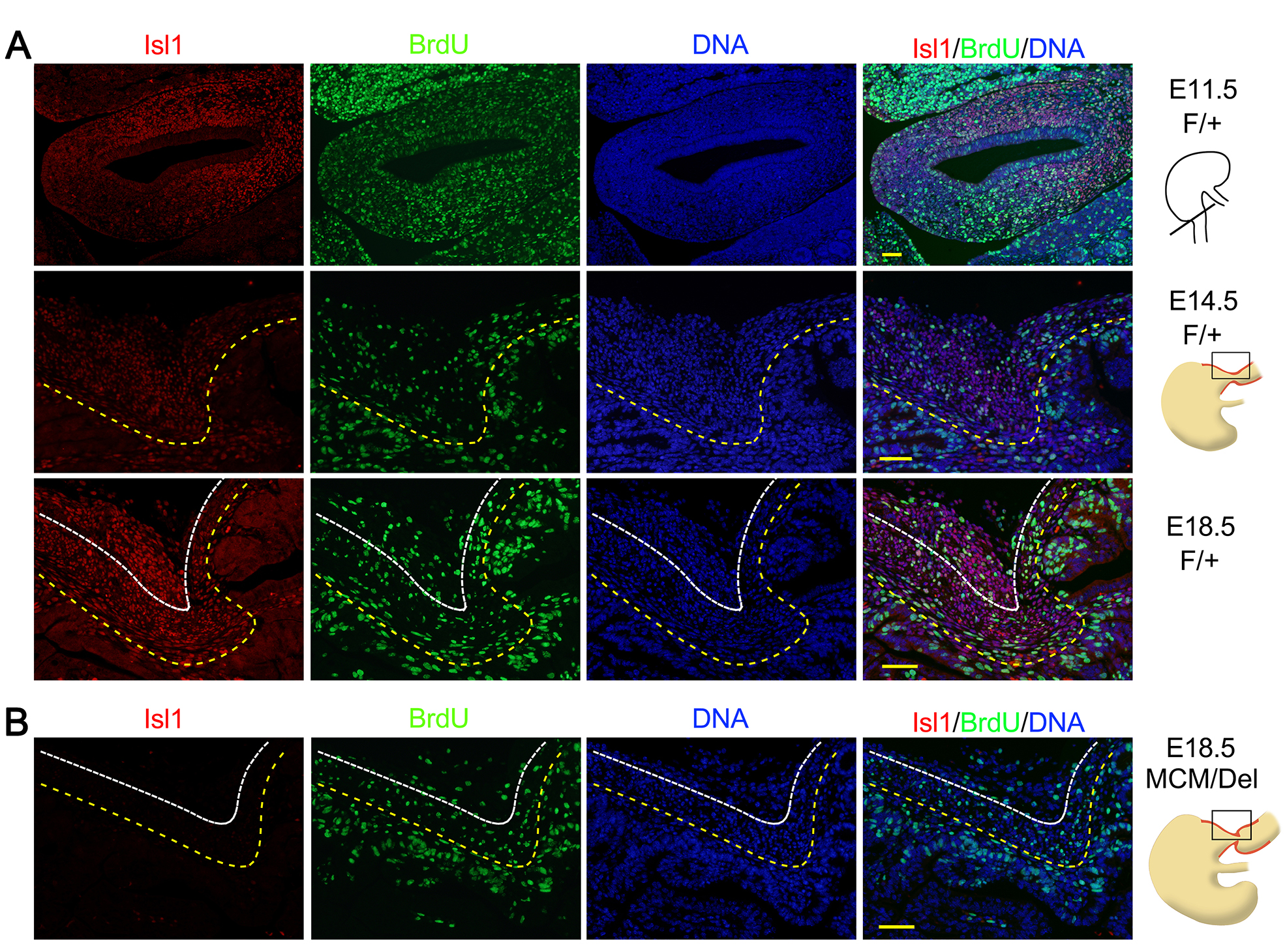


**Figure S9: Double immunostaining for Isl1 and BrdU in smooth muscle layer of the pylorus.** (**a**) Isl1 and BrdU co-expression in smooth muscle cells at E11.5, E14.5 and E18.5 in *Isl1^F/+^* mice. (**b**) Isl1 and BrdU dual-immunostaining at E18.5 in the pyloric ICM and OLM layer of *Isl1^F/+^* and *Isl1^MCM/Del^* *mice*. Cell proliferation was not significantly changed in pyloric ICM and OLM layer of *Isl1^MCM/Del^* mutants when compared to *Isl1^F/+^* controls. Yellow dotted lines mark the epithelial basement membrane and white dotted lines indicate ICM and OLM boundary. Red staining is Isl1, green staining is BrdU, and DAPI nuclear counterstaining (DNA) is blue. Scale bars: 50 μm.


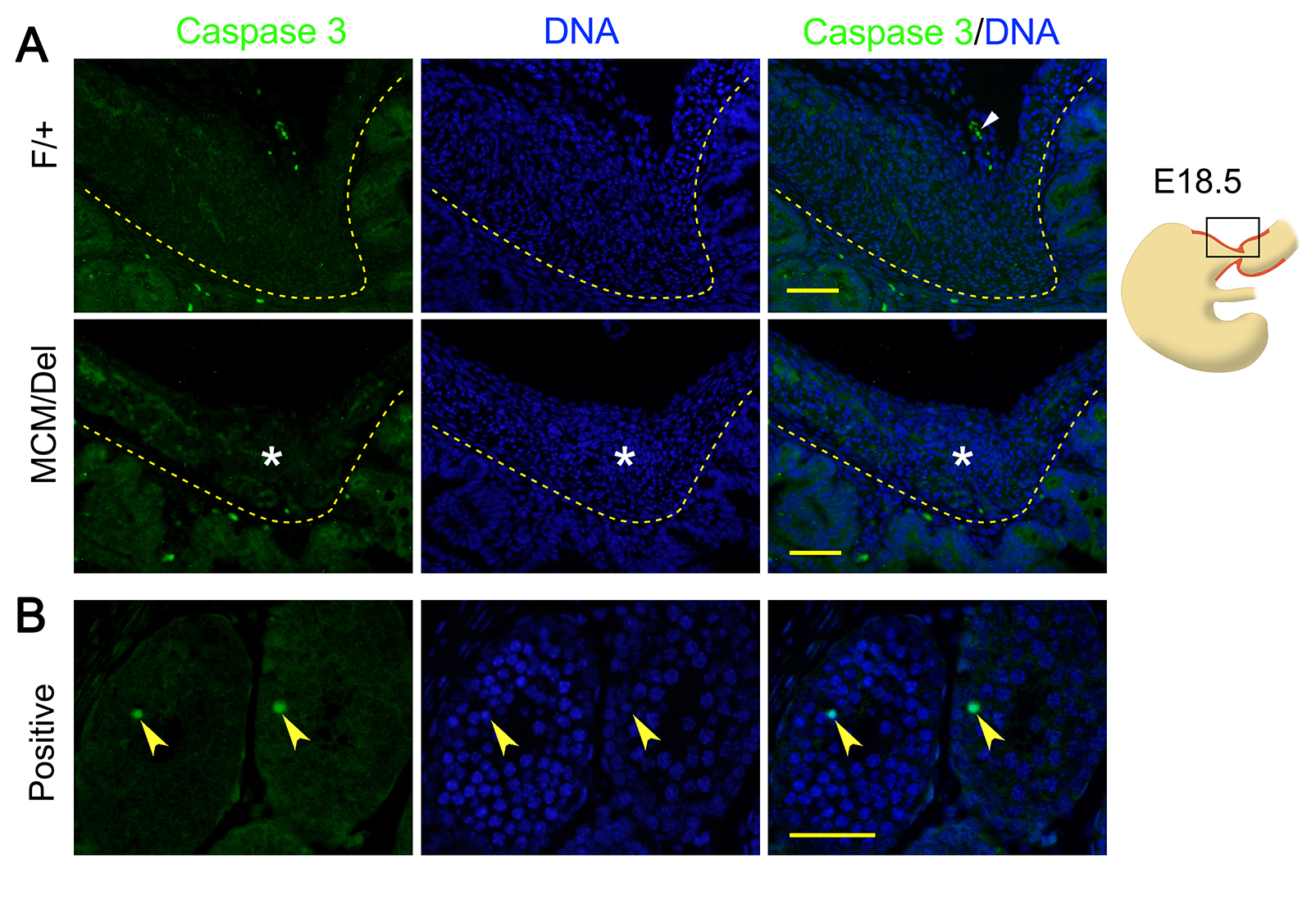


**Figure S10: No difference in smooth muscle cell apoptosis in *Isl1^MCM/Del^* mutants.** (**a**) Caspase 3 immunofluorescence at E18.5 in *Isl1^F/+^* and *Isl1^MCM/Del^* pylorus. No Caspase 3 positive cells were observed in the pyloric smooth muscle layer of *Isl1^MCM/Del^* mutants and *Isl1^F/+^* control (asterisk regions). Yellow dotted lines mark the epithelial basement membrane. White arrowhead indicates non-specific stain. Scale bars: 50 μm. (**b**) Adult testis sections were used as positive control. Yellow arrowheads shows representative Caspase 3 positive cells. Green staining is Caspase 3, and DAPI nuclei counterstaining (DNA) staining is blue. Scale bars: 50 μm.
