# Supplementary material for: LIM homeodomain transcription factor Isl1 directs normal pyloric development by targeting Gata3
Source: BMC Biol. 2014 Mar 27;12:25. doi: 10.1186/1741-7007-12-25 (PMC4021819; doi:10.1186/1741-7007-12-25)
Supplement: Additional file 2 — Supplementary Information. This file contains Tables S1 to S4. [file 1741-7007-12-25-S2.doc]

**Table S1:** Sequences of primers for PCR, semi-quantitative PCR and RT-qPCR

| **Gene** | **Primer sequence 5’ -3’** | **Accession number** |
| --- | --- | --- |
| **α-SMA** | fw- TCAGGAACCCTGAGACGCTGCT | NM_007392.3 |
|  | rev- CCAGCGAAGCCGGCCTTACAG |  |
| **Bapx1** | fw- AGGCGAGGATGACAGCGTTAG | NM_007524.3 |
|  | rev- ACCTGGGCGTGCGAGAA |  |
| **Barx1** | fw- TAGTGCTGCAAGGTGGCGGC | NM_007526.4 |
|  | rev- TCGCAATTTCGGTCGCTGGGC |  |
| **Gata3** | fw- AGGCAACCACGTCCCGTCCT | NM_008091.3 |
|  | rev-TACCTGGCTCCCGTGGTGGG |  |
| **Gremlin** | fw- GGCTGGAGGCTGCTTATG | NM_011824.4 |
|  | rev- CTGCCCCTGTGGATGTTT |  |
| **Nkx2.5** | fw- CCGCCAACAGCAACTTCG | NM_008700.2 |
|  | rev-CGGGCTCTTTCCCTACCAG |  |
| **Six2** | fw- TGGGAGGGCGATAGATTC | NM_011380.2 |
|  | rev- AAGGATACCGAGCAGACCAT |  |
| **Gapdh** | fw- AAGCCCATCACCATCTTCCAG | NM_008084.2 |
|  | rev-AGGGGCCATCCACAGTCTTCT |  |
| **Exon 3/6** | fw- CCCTCTCAGTCCCTTGCATC | NM_021459.4 |
|  | rev- CATGCTGTTGGGTGTATCTG |  |
| **MCM** | fw- ACTATTTGCCACCTAGCCACAGCA |  |
|  | rev-GTTCAGCATCCAACAAGGCACTGA |  |
| **Isl1F/+** | fw- GGTCTCTGGAACATCCCACATTGT |  |
|  | rev- CTGTTCCTACTCCCCATTCACT |  |

Primers sequences for detecting Isl1 knockdown efficiency and identifying gene expression change in *Isl1^MCM/Del^* mouse embryos.

**Table S2:** Sequences of probe and plasmid construction primers

| **Gene** | **Primer sequence 5’-3’** | **Accession number** | |
| --- | --- | --- | --- |
| **Gata3** | fw- GTCCTCATCTCTTCACCTTCCC | | NM_008091.3 |
|  | rev- CATTAGCGTTCCTCCTCCAG | |  |
| **Nkx2.5** | fw- CCACGCGTGCTCTTCTCGCA | | NM_008700.2 |
|  | rev- GGTGGGCGACGGCAAGACAA | |  |
| **Gremlin** | fw- GCCACACCCAAACCACCCGA | | NM_011824.4 |
|  | rev- CAGCCTGACCGTTCCTGCGTC | |  |
| **Isl1** | fw- CAGCAAGAACGACTTCGTGA | | NM_021459.4 |
|  | rev- GGACTGGCTACCATGCTGTT | |  |
| **Gata3-P1 WT** | fw- CGAGCTCTCCTCTTTCCCAATTACCCA | | NT_039202.8 |
|  | rev- CCCAAGCTTAATATCTAATAGGCCCCTCC | |  |
| **Gata3-P6 WT** | fw- CGAGCTCCTCTACTGGGCGTCTTCCAG | | NT_039202.8 |
|  | rev- CCCAAGCTTGCTACTTTATTCTGCTTTCACCCG | | |

Primers sequences for producing ISH probes and constructing plasmid for Luciferase assays.

**Table S3:** Sequences of primers used for ChIP assays

| **Primers name** | **Primer sequence 5’-3’** |
| --- | --- |
| **P1** | fw- GCCATCAAAAGAGCAACG |
|  | rev- TAATTCAATCACCAGAGGAGC |
| **P2** | fw- CTCCATCCCCACTACTACA |
|  | rev- CTGACGCCAAGAACTGAA |
| **P3** | fw- CGTCAGCAGCTTTCTTTC |
|  | rev- TCAGTCAGCCAGTCCCTA |
| **P4** | fw- CTGGCTGACTGATGCCTTAT |
|  | rev- CGAGTCTTCCCTCCCTTC |
| **P5** | fw- CAGGGTGAACTCAGAAGG |
|  | rev- GCTTTAGAGGGTTACTGGA |
| **P6** | fw- CGATGATTGGTCCTTACA |
|  | rev- TCGGGGTGCTACTTTATT |
| **P7** | fw- GTCACTAAGGTCAAAAGCAC |
|  | rev- TGGCGAAGTAACTGAAGAT |
| **P8** | fw- GCTGGTTCGGAGGCAAAT |
|  | rev- AGGGGACAGGCAGGAGGGA |
| **P9** | fw- CTTGGAGGGACTGTTTGTG |
|  | rev- GGAGCCCAGGACTGACTAA |
| **P10** | fw- TCCTCCCTAAACCCTCCT |
|  | rev- GTCCGCAGTCACCTCCAT |
|  |  |

Primers of ten areas that contain putative Isl1 binding sites located at upstream of *Gata3* (NT_039202.8).

| Antibody | Blocking media | Primary antibody | Secondary  Antibody | Tertiary  Antibody |
| --- | --- | --- | --- | --- |
| Isl1  Cdx2 | 1 hour; RT | Overnight at 4°C; RT | 2 hours; RT | 2hours; RT |
| α-SMA  Gata3  PGP9.5  Sox9  BrdU  Caspase3  Pdx1 | 1h; RT | Overnight at 4°C; RT | 2 hours; RT | N/A |

**Table S4:** Immunofluorescence and Immunohistochemistry protocols.

RT =room temperature
